# Supplementary material for: Milk and resistance exercise intervention to improve muscle function in community-dwelling older adults at risk of sarcopenia (MIlkMAN): protocol for a pilot study
Source: BMJ Open. 2019 Oct 8;9(10):e031048. doi: 10.1136/bmjopen-2019-031048 (PMC6797244; doi:10.1136/bmjopen-2019-031048)
Supplement: Supplementary data [file bmjopen-2019-031048supp001.pdf]

SUPPLEMENTARY MATERIAL

Appendix 1

THE SARC-F QUESTIONNAIRE[30]

Strength: How much difficulty do you have in lifting and carrying 10 pounds / a bag of shopping?
None: 0
Some: 1
A lot or unable: 2

Assistance in walking: How much difficulty do you have walking across a room?
None: 0
Some: 1
A lot, use aids, or unable: 2

Rise from a chair: How much difficulty do you have transferring from a chair or bed?
None: 0
Some: 1
A lot or unable without help: 2

Climb stairs: How much difficulty do you have climbing a flight of 10 stairs?
None: 0
Some: 1
A lot or unable: 2

Falls: How many times have you fallen in the past year?
None: 0
1 to 3 falls: 1
4 or more falls: 2

Appendix 2

CR100 scale[49]

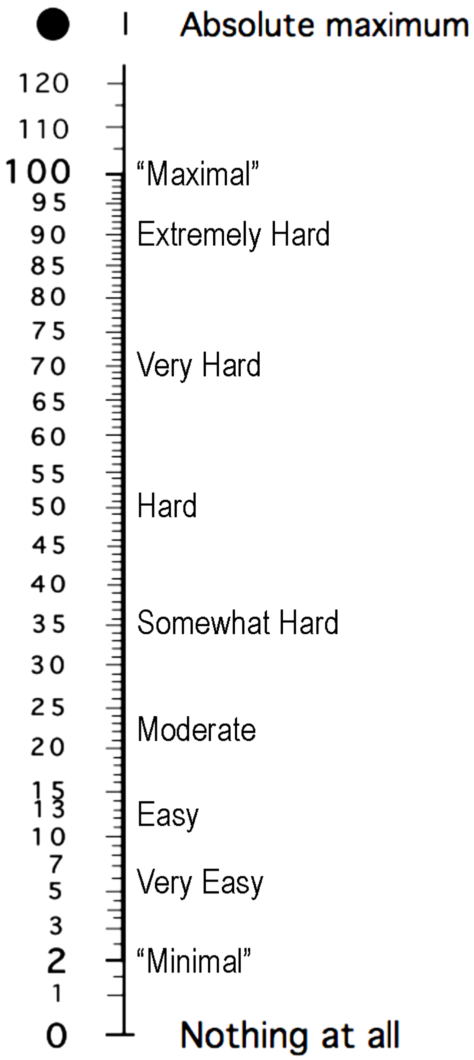

Instructions for participants (explained to participants by exercise physiologist)
Assessment ~10 minutes after exercise

- Using the scale, we would like you to rate your perceptions of EFFORT (RPE), that is, how difficult the session felt to you.
- Your perception of EFFORT should be a conscious awareness of how hard (or easy) the whole session was. It should not be influenced your feelings of fatigue, pain or discomfort (try separating these from effort as best you can).
- You will be asked to rate your perceptions of overall effort (the whole session after the warm-up), and then separate scores for upper-body muscle effort (arm muscles) and lower-body muscle effort (leg muscles).

Appendix 3

Visual analogue scale for muscle soreness

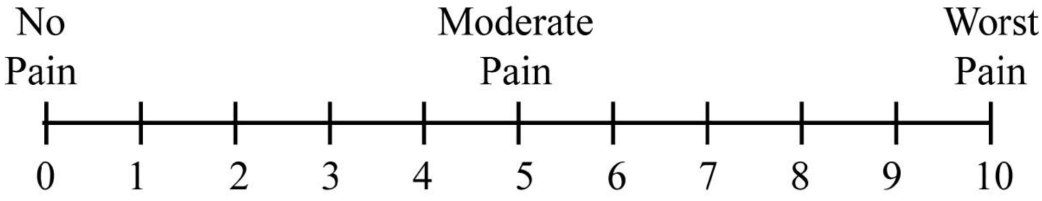

0 = no pain, 1-3 = mild pain, 4-6 = moderate pain; 7-10 = severe

Instructions for participants (explained to participants by a researcher)

- This is a Visual Analogue Scale. The scale describes the intensity of your muscle soreness, 0 meaning no pain at all, 1 to 3 meaning mild pain, 4-6 moderate pain, and 7-10 the worst pain.
- Assessment ~45 minutes after exercise: Which number on the scale describes the best your muscle soreness in your arms and legs?
- *Assessment in the evening over the telephone (6-7 hours after exercise)*: A researcher will call you in the evening after each visit to the gym to ask you again about your muscle soreness in your arms and legs. Please use this visual analogue scale to rate your muscle soreness in your arms and legs.
