# Supplementary material for: Milk and resistance exercise intervention to improve muscle function in community-dwelling older adults at risk of sarcopenia (MIlkMAN): protocol for a pilot study
Source: BMJ Open. 2019 Oct 8;9(10):e031048. doi: 10.1136/bmjopen-2019-031048 (PMC6797244; doi:10.1136/bmjopen-2019-031048)
Supplement: Author's manuscript [file bmjopen-2019-031048.draft_revisions.pdf]

BMJ Open

BMJ Open is committed to open peer review. As part of this commitment we make the peer review history of every article we publish publicly available.

When an article is published we post the peer reviewers' comments and the authors' responses online. We also post the versions of the paper that were used during peer review. These are the versions that the peer review comments apply to.

The versions of the paper that follow are the versions that were submitted during the peer review process. They are not the versions of record or the final published versions. They should not be cited or distributed as the published version of this manuscript.

BMJ Open is an open access journal and the full, final, typeset and author-corrected version of record of the manuscript is available on our site with no access controls, subscription charges or pay-per-view fees (http://bmjopen.bmj.com).

If you have any questions on BMJ Open's open peer review process please email info.bmjopen@bmj.com

BMJ Open

Milk and resistance exercise intervention to improve muscle function in community-dwelling older adults at risk of sarcopenia (MIlkMAN): protocol for a pilot study

Journal:	*BMJ Open*
Manuscript ID	bmjopen-2019-031048
Article Type:	Protocol
Date Submitted by the Author:	15-Apr-2019

Complete List of Authors:	Granic, Antoneta; Newcastle University, Institute of Neuroscience<br>Hurst , Christopher; Newcastle University, Institute of Neuroscience<br>Dismore , Lorelle ; Newcastle University, Institute of Neuroscience<br>Davies , Karen ; Newcastle University, Institute of Neuroscience<br>Stevenson , Emma ; Newcastle University, Institute of Cellular Medicine<br>Sayer, Avan; Newcastle Upon Tyne Hospitals NHS Foundation Trust, NIHR Biomedical Research Centre; Newcastle University<br>Aspray , Terry ; Newcastle University, Institute of Cellular Medicine

Keywords:	pilot study, older adults, sarcopenia, physical function, whole milk, resistance exercise

SCHOLARONE™
Manuscripts

Milk and resistance exercise intervention to improve muscle function in community-dwelling older adults at risk of sarcopenia (MIlkMAN): protocol for a pilot study

Antoneta Granic[1-3*], Christopher Hurst[1,2†], Lorelle Dismore[1,2†], Karen Davies[1-3], Emma Stevenson[4,5], Avan A Sayer[1-3], Terry Aspray[1,2,4, 6]

[1]AGE Research Group, Institute of Neuroscience, Newcastle University, Newcastle upon Tyne, United Kingdom

[2]NIHR Newcastle Biomedical Research Centre, Newcastle upon Tyne Hospitals NHS Foundation Trust and Newcastle University, Newcastle upon Tyne, United Kingdom

[3]Newcastle University Institute for Ageing, Newcastle upon Tyne, United Kingdom

[4]Institute of Cellular Medicine, Newcastle University, Newcastle upon Tyne, United Kingdom

[5]Human Nutrition Research Centre, Newcastle University, Newcastle upon Tyne, United Kingdom

[6]Newcastle upon Tyne Hospitals NHS Foundation Trust, Musculoskeletal Unit, Freeman Hospital, Newcastle upon Tyne, United Kingdom

[†]equal contribution

For peer review only

*correspondence:

E-mail: antoneta.granic@newcastle.ac.uk

Phone: +44 (0) 1912081112

Biomedical Research Building, 1st Floor

Campus for Ageing and Vitality

Newcastle University

Newcastle upon Tyne, NE4 5PL

United Kingdom

Word count: 4,230

For peer review only

ABSTRACT

Introduction: Sarcopenia is a progressive muscle disorder characterised by decline in skeletal muscle mass, strength and function leading to adverse health outcomes, including falls, frailty, poor quality of life, and death. It occurs more commonly in older people and can be accelerated by poor diet and low physical activity. Intervention studies incorporating higher dietary protein intakes or protein supplementation combined with resistance exercise (RE) have been shown to limit muscle function decline. However, less is known about the role of whole foods in reducing the risk of sarcopenia. Milk is a source of high-quality nutrients, which may be beneficial for skeletal muscle. This pilot study examines the feasibility and acceptability of milk consumption with RE to improve muscle function in community-dwelling older adults at risk of sarcopenia.

Methods and Analysis: 30 older adults aged ≥65 years will be randomly allocated to three groups: 'whole milk + RE', 'skimmed milk + RE' or 'control drink + RE'. Assessments will take place in participants' homes, including screening (milk allergies, grip strength, walking speed), baseline and post-intervention health and function. All participants will undertake a structured RE intervention twice/week for 6 weeks at a local gym, followed by the consumption of 500ml of whole or skimmed milk (each ~20 g of protein) or an isocaloric control drink and another 500ml at home. Participants' views about the study will be assessed using standardised open-ended questions. The primary outcomes include feasibility and acceptability of the intervention with recruitment, retention, and intervention response rates. Analyses will include descriptive statistics, exploration of qualitative themes and intervention fidelity.

Ethics and dissemination: The North East–Newcastle and North Tyneside Research Ethic Committee 1 (18/NE/0265) approved the study. Outputs include pilot data to support funding applications; public involvement events; presentation at conferences, and peer-reviewed publication.

Trail registration number: ISRCTN13398279; Pre-results.

For peer review only

Keywords: pilot study, older adults, physical function, muscle strength, sarcopenia, whole milk, resistance exercise, dietary protein, quality of life

Strengths and limitations of this study

- This pilot study will examine the feasibility and acceptability of milk in combination with resistance exercise (RE) as an intervention for maintenance of muscle health in community-dwelling older adults who may be at risk of sarcopenia (aged ≥65).
- The study will examine intervention fidelity (2 × 500ml (~40g of protein) of whole and skimmed milk after structured RE twice/week over 6 weeks) in the community, and participants' attitudes about the study.
- The study will provide quantitative and qualitative evidence to support planned future research, but has limited statistical power to detect differences in muscle functioning between the groups pre and post intervention.

peer review only

INTRODUCTION

The UK population is ageing rapidly; the number of adults aged ⩾65 increased by 17.3% in the last decade, and in mid-2017 was estimated to account for 18.2% of the total population of 66 million[1]. Understanding factors associated with healthy ageing[2] such as diet and physical activity[3] for optimising health and wellbeing of an ageing population is essential for the development of effective interventions.

Sarcopenia is a progressive, generalised muscle disorder characterised by decline in skeletal muscle mass, strength and function[4], which leads to an increased risk of falls, frailty, disability, low health-related quality of life (QoL) and death[5-8]. It occurs more commonly in older people and can be accelerated by poor diet and low physical activity[8,9]. The prevalence of sarcopenia increases with advancing age, with more than 20% of men and women aged ≥85 years affected[10], which results in an estimated excess of health care cost of £2.5 billion/year in the UK[11]. This emphasises the need for sustainable preventive measures aimed to preserve and optimise muscle health and function in a rapidly ageing population.

Protein intake and exercise for healthy muscle ageing

Loss of muscle mass and strength can be accelerated by poor diet, low levels of physical activity and the presence of long-term conditions[8,9,12], leading to diminished QoL[7,13]. Adequate intake of dietary protein and resistance exercise (RE) are recognised as effective interventions to promote skeletal muscle health and reduction of physical decline[14,15]. Specifically, intervention studies that examined a combined effect of protein supplements, comprising essential amino acids (EAA) and RE to stimulate muscle protein synthesis (MPS) have observed an increase in total muscle protein within 3-5 hours following exercise in both young and older adults[16]. Compared with young adults, older adults experience a blunted response after protein ingestion to stimulate MPS (anabolic resistance), especially in response to lower amounts of protein or EAA of <20g or <10g, respectively. Other studies have shown that greater amounts of protein supplementation and intermittent feeding in

For peer review only

combination with repeated bouts of RE resulted in increased muscle mass in older adults, even in those diagnosed with frailty and sarcopenia[17-19].

However, there is limited research on the role of whole foods rich in protein (e.g. milk and dairy products, fish, and meats) in maintaining skeletal muscle mass, strength and function in older adults at risk of sarcopenia. Regular consumption of high-quality, nutrient-dense foods, high in macro- and micronutrients relevant for muscle[20] within a varied diet may provide a platform for developing strategies for maintenance of muscle health and function in later life that do not include supplements and medical products, and may be easier adopted as a behavioural change in older adults[21].

Milk for muscle health: current evidence and why this pilot is needed

Cow's milk is an example of a whole food with the potential to ameliorate loss of skeletal muscle mass, strength and function. Whole milk (3.6% fat) is a source of high-quality proteins (whey and caseins), minerals (e.g. calcium, phosphorus, magnesium), vitamins (e.g. A, B, D, and E), carbohydrates, bioactive lipids and fatty acids (mono- and polyunsaturated, and saturated fatty acids)[22]. Whey protein is considered superior to other protein sources for MPS after exercise in younger and older adults because of its greater bioavailability and solubility, and higher content of the branched-chain amino acids, including leucine[23-25]. Furthermore, the concurrent intake of milk fats with protein in whole milk has been shown to increase the use of EAA for MPS after exercise in young men compared with skimmed milk (0.3% fat)[26], suggesting additional benefits of milk lipids for muscle. Other benefits of milk containing fat include reduction in exercise-related muscle damage, soreness, and decline in muscle performance in young adults and athletes[27,28] compared with energy–matched (isocaloric) carbohydrate drink. However, little is known about the effect of milk and protein-fat ratio in milk on muscle in older adults, particularly the impact on muscle function of varying milk fat contents (whole versus skimmed) providing >20g protein/day after exercise.

For peer review only

We hypothesised that whole milk (3.6% fat), providing >20g of protein and the same amount of energy as fat and protein-free carbohydrate drink, after structured exercise conducted in the community may be a feasible and acceptable intervention for maintaining skeletal muscle mass, strength and function in older adults at risk of sarcopenia.

Study aims

The primary aims are:

(1) To examine the feasibility and acceptability of whole (3.6% fat) or skimmed milk (0.3% fat) in combination with RE as an intervention in community-dwelling older adults aged ≥65 at risk of sarcopenia. This aim will answer the following questions: Is an intervention of 2 × 500ml milk + RE twice/week for 6 weeks (a) feasible and (b) acceptable to older adults?

(2) To provide essential data for planned future substantive research.

The secondary aim of the study will be to explore whether consumption of whole or skimmed milk + RE has an influence on physical performance, muscle mass, strength and self-reported QoL in older adults at risk of sarcopenia.

METHODS AND ANALYSIS

Study design

This is a pilot study involving 30 participants (aiming for 15 men and 15 women) aged ≥65 who will be randomised into three intervention groups: (group 1) 'whole milk + RE'; (group 2) 'skimmed milk + RE', and (group 3) 'control drink' + RE. Data will be collected from: (1) health and functioning assessments (screening, baseline and post-intervention interview); (2) the nutrition + exercise intervention over 6 weeks, and (3) participants' feedback about the study.

For peer review only

Exclusion and inclusion criteria

The study will include older adults who are registered patients with General Practitioner (GP) practices within the National Institute for Health Research (NIHR) North East and North Cumbria Clinical Research Network (CRN), United Kingdom. Table 1 lists the inclusion and exclusion criteria that are applied to patient database searches, performed in GP practices and screening interviews conducted by the research team.

Table 1. Inclusion and exclusion criteria for the MIlkMAN: pilot

Criteria	Patient database searches	Screening interview
Inclusion	aged 65 and over	
	live in the community	
Exclusion	diabetes mellitus type 1 or type 2	lacks capacity to consent to participate
	chronic kidney disease stage 4 or 5 (estimated glomerular filtration rate <30ml / min /1.73m^2)	lactose intolerance
	liver function impairment (AST >2.5 times upper limit of normal range within the last 6 months)	dislikes milk or cranberry juice (control drink)
	chronic lung disease requiring maintenance steroid therapy (e.g. COPD, severe asthma)	participated in a structured RE training and gym programme in the last month
	end-stage terminal illness	dislikes gym exercise with equipment
	cardiac pacemaker or severe heart failure or other significant heart disease	unintentional weight loss ⩾5kg in the last 3 months
	uncontrolled hypertension (>160/100 mmHg) and uncontrolled hypotension (<100 mmHg systolic) within last 6 months	unable to understand instructions for muscle strength and function assessments in English or unwilling to participate in protocol when explained
	hip or knee replacement	an individual who the research team (exercise physiologist) evaluates as not suitable for the intervention because of safety reasons
	impaired mobility (unable to walk without an aid including wheelchair	
	current prescription of warfarin (potential interference with control drink)	
	BMI ⩾30kg/m2	
	an individual who the GP feels it is inappropriate for the research team to	

	approach for safety reasons: any medical and physical conditions that preclude safe participation in a RE programme (long-term conditions likely to lead impaired function over 6 months)

AST, Aspartate Aminotransferase; BMI, body mass index; COPD, Chronic Obstructive Pulmonary Disease; GP, general practitioner.

Study population and recruitment

Primary care recruitment will be carried out with the assistance of North East and North Cumbria CRN, England, which provides support with access to general practices and their patients. Two practices in North Tyneside Clinical Commissioning Group were identified for feasibility using exclusion/inclusion criteria (Table 1, left column) and provided feedback during the application for funding in 2018.

Recruitment will be organised in two stages: pre-screening (GP practices) and screening (research team). At the pre-screening stage, practices will identify potential participants from their patient database using exclusion/ inclusion criteria (Table 1, left column), and then mail out recruitment packs, containing detailed information about the study with a reply slip. Interested individuals will be interviewed over the telephone by a researcher using a 5-item SARC-F questionnaire (Appendix 1)[29] to assess any difficulties with day-to-day activities (lifting and carrying 10 pounds, walking, rising from a chair and climbing stairs), and number of falls in the past year. Those evaluated by the research team to have no major difficulties that would preclude safe participation in the exercise programme (e.g. unable to walk across a room), will be visited in their own home for a screening interview to obtain written informed consent, and to evaluate inclusion/exclusion criteria not screened through GPs (Table 1, right column). Participants' muscle strength (grip strength, GS) and function (walking speed) will also be determined based on the establish cut-offs (low grip GS: <20 kg (women), and <30 kg (men)[30]; low walking speed: <0.8 m/s or ⩾5 s over 4 m distance[30]). GS measurements (high or low) will be used for minimisation along

For peer review only

with sex to allow equal distribution of those with muscle strength weakness across the intervention groups.

Study outcomes

Table 2 lists the primary and secondary study outcomes and when they are completed.

Table 2. Study outcome measures

Measure	Screening	Baseline	Post-intervention
Primary			
Feasibility and acceptability of intervention in a local gym setting			×
Applicability			×
Dosage and duration of intervention			×
Compliance		×	×
Attrition		×	×
Adverse health effects			×
Response rates to questionnaires, assessments, and intervention	×	×	×
Secondary			
Short Physical Performance Battery[31] (balance, 4m-gait speed, 5-chair stands)		×	×
Muscle mass[32]		×	×
Grip strengh[33]	×	×	×
SF-12 Health Survey[34]		×	×
Barthel Index[35]		×	×

The secondary outcome measures will explore differences in physical performance measures between the groups, pre and post-intervention. GS (muscle strength)[33] will be expressed as the maximum reading of 6 trials of both hands using a Jamar hand-held 5030J1 dynamometer. Body composition (muscle mass) will be measured using Bioelectric Impedance Analysis[32] (BIA; Tanita MC-780MA Body Composition Analyzer). Self-reported QoL will be measured using SF-12[34], and activities of daily living with Barthel Index[35].

Randomisation

Thirty participants will be allocated to one of the three interventions to ensure balanced allocation of participants between the groups based on sex and muscle strength (GS at screening assessment) using a free, open-source minimisation software (MiniPy 0.3, http://minimpy.sourceforge.net)[36]. The software features elements of randomness in the

For peer review only

minimisation algorithm by allocating the first participant randomly into one of the interventions, and assigning the subsequent participants on hypothetical stepwise allocation to every group and computation of the imbalance score corresponding to each allocation. The imbalance scores are compared and participants allocated to the group corresponding to the least imbalance score (preferred group).

Consent

Written informed consent will be obtained by a researcher visiting the participants during the home-based screening assessment prior to randomisation. Capacity to consent will be assessed using an established consent pathway. Throughout the active research phase (i.e. from baseline to post-intervention assessment), the notion of process consent will be implemented, requiring an ongoing exchange of information about the study and confirming the participants willingness to proceed, ensuring that participants are free to reconsider and withdraw from the study at any time. If a participant loses capacity during the research process, he/she will be withdrawn from the study.

Flow diagram of the study

A flow diagram of the study protocol with timelines is outlined in Figure 1. The recruitment (assessment of eligibility) up to allocation (randomisation into 3 groups) will be finalised within 3 months, followed by baseline assessments for health and functioning in participants' homes, and a 6-week intervention in a local gym.

>>Insert Figure 1<<

Figure 1. Study flow chart.

Home-based post-intervention assessments, including participants' feedback about the study, will be conducted the week following completion of the intervention, and finalised within 3 weeks. Data analysis will be completed after active data collection (from randomisation to post-intervention assessment). Data collection for each participant will span

For peer review only

approximately 10 weeks: (1) week 1: consent and home-based screening assessment; (2) week 2: home-based baseline interview; (3) week 3 to week 8: 6-week intervention twice/week (12 visits at a local gym/sport centre); (4) week 10: post-intervention home-based interview. Except for the intervention (6 consecutive weeks), this time scale can be adjusted to participants' individual needs with a maximum 3 weeks gap between baseline assessment and the first week of intervention, and a maximum 3 weeks gap between the last week of intervention and post-intervention assessment.

Data collection

To determine the feasibility and acceptability of the study, the following data will be collected and analysed. The number of individuals approached; the reasons for not opting to take a part in the study (reported with permission); the recruitment and retention rates; the reasons for attrition; the completion of objective assessments and questionnaires; the number of RE sessions completed, and compliance with the milk/control drink intake. Other health and functioning data will be collected at the home-based screening and baseline interview, during the intervention (at the gym), and at the home-based post-intervention interview. Participants' attitudes and opinions about the study will be collected at the post-intervention interview using a combination of multiple-response and standardised open-ended questions.

Screening interview

Once potential participants have been identified by the GP practices, having expressed an interest in participation and being interviewed over the phone by a researcher, a mutually convenient appointment will be arranged for a screening visit at the participant's home. Participants will be screening for other exclusion/ inclusion criteria not assessed at by the GP practices (Table 1, right column), and to establish participants' muscle strength (GS) and functioning status (walking speed) based on the established cut-offs[30]. Informed consent will be obtained before any assessment is undertaken. Eligible individuals will be informed

For peer review only

about the study procedure and their journey through the study (from randomisation to post-intervention assessment).

Baseline interview

Table 3 lists domains and assessments for the baseline interview with times needed to administer (in minutes). The detailed health and functioning profile will involve minimal risk and inconvenience to participants, and it will be conducted within 70 minutes (including breaks and excluding assessments done at the gym).

For peer review only

Table 3. Domains and assessments at baseline

**Domain and assessment**	**Time to administer (min)**
Sociodemographic profile	total: 6
age	
sex	
marital status	
education	
social class (NS-SEC)[37]	
deprivation (Multiple Index of Deprivation)[38]	
General health	total: 54
SF-12 Health Survey[34]	4
self-reported diseases diagnosed by a doctor	2
list of medication (prescribed and over-the-counter)	2
Mini Mental State Examination	10
Geriatric Depression Scale (15-item version)[39]	7
Barthel Index (Activities of Daily Living)[35]	3
blood pressure (systolic and diastolic)*	5
Intake24: 24-hr dietary recall[40]* (https://intake24.co.uk/)	20
appetite (a 4-item Simplified Nutritional Appetite Questionnaire)[41]*	1
Lifestyle	total: 5
self-reported physical activity[42]	3
smoking status	1
alcohol intake	1
Anthropometry	total: 14
demi-span	2
waist and hip circumference	3
calf circumference	2
muscle mass (body composition by BIA)[32]*	7
Physical functioning	total: 24
Short Physical Performance Battery (SPPB)[31]	10
balance (a side-by-side tandem; semi-tandem; tandem)	3
4-m gait speed	3
5-chair stands	4
maximum grip strength (measured 3 times in each arm)	4

*Assessments done at the intervention site (gym) before and after each RE session (blood pressure), and before the first RE session (diet, appetite, body composition).

For peer review only - http://bmjopen.bmj.com/site/about/guidelines.xhtml

Intervention

Resistance exercise (RE)

All participants will perform two RE sessions per week for 6 weeks at a community leisure centre (The Parks, North Tyneside Council, North Shields, UK). Each RE session will be ~45-60 min in duration, with a minimum of 48 hours between sessions, and will be completed in groups of 2-4 participants under the supervision of an experienced exercise physiologist (CH). Exercise intensity, volume, frequency and duration have been determined based on recent literature[43-45] and the American College of Sports Medicine (ACSM) recommendations for older adults[44]. With the exception of the structured exercise sessions and the nutritional intervention prescribed, participants will be asked to maintain their usual diet, level of physical activity and lifestyle throughout the duration of the intervention period.

During the first RE session participants will be familiarised with the exercises (leg press, leg curl, seated row, chest press) as well as the equipment to be used throughout the intervention with correct technique demonstrated and extensively described. Following this, participants' one repetition maximum (1RM) will be estimated for all four exercises using a previously established equation[46].

Following the initial RE session, each remaining session will begin with a 5-minute warm-up performed at progressive intensity using either a cycle ergometer or treadmill. Participants will then complete 2-4 sets of 8-12 repetitions at a workload of 70-79%1RM[43,44] for all four of the exercises listed above. Each session will conclude with a short cool-down period of low intensity aerobic exercise. In an attempt to promote participants' engagement with RE, each will receive a booklet with diagrams and short instructions with space to record the details of the exercises they have successfully completed. Participants' gym attendance, sets and repetitions completed, and weight lifted will be recorded throughout the intervention.

Blood pressure and heart rate will be measured pre and post each RE session in each participant and compared to the guidelines provided by the American College of

For peer review only

Cardiology/American Heart Association Task Force[47] and existing literature[48,49]. Using the CR100® scale[50] (Appendix 2), participants will provide an overall session rating of perceived exertion (sRPE) as well as differential ratings of perceived exertion for upper-body muscle exertion (RPE-U) and lower-body muscle exertion (RPE-L) approximately 10 minutes after the completion of each RE session. Muscle soreness will be assessed using a simple visual analogue scale (Appendix 3) at ~40-45 minutes and at ~6-7 hours after each RE session.

Nutritional intervention

On average, 500ml milk contains ~20g of protein needed to stimulate MPS above stimulation provided by RE[16,17]. Whole cow milk (nutritional estimates of 22 UK samples during winter and summer) provides 66 kcal/100g of energy[51]. Arla Cravendale® whole milk contains 3.6g fat, 3.4g protein, and 4.7g of carbohydrates per 100g of milk. Arla Cravendale® skimmed milk contains 0.3g fat, 3.6g protein, and 4.9g of carbohydrate per 100g of milk. The energy content of the control drink (cranberry juice; Ocean Spray Classic®; 23/kcal/100g of energy) will be balanced to match whole milk energy content and supplemented with maltodextrin (4kcal/g; www.myprotein.com) on the day of intervention. Both milk and juice will be provided in packs of 1L, bought fresh on a weekly basis through a local retailer and kept in a locked refrigerator at the Campus for Ageing and Vitality, Newcastle University.

The milk/control drink will be consumed as a bolus intake of 500ml under the supervision of a researcher immediately after exercise during the recovery period, aiming for complete consumption within ~45 minutes prior to leaving the centre. The second dose of 500ml will be consumed at the participants' home over the next 4-5 hours as a part of their usual diet with other foods. Participants' compliance with consumption of the milk/control drink will be checked in the evening (~6-7 hours post-exercise). Each participant will be provided with a plastic measuring jug (500ml) to measure their consumption at home and to report it back to a researcher over the telephone.

For peer review only

Post-intervention interview

Table 3 lists the domains and assessments that will be repeated at the post-intervention interview. Briefly, a home visit will be arranged with each participant after the 6-week intervention to assess his/her general health and physical functioning, including SPPB, muscle mass (body composition), muscle strength (GS), SF-12, Barthel Index, diet and appetite. Additionally, participants' feedback will be collected at the end of the post-intervention interview using a combination of structured multiple-response and standardised open-ended questions. The following themes will be explored: (1) attitudes and barriers to consuming of 2 × 500ml milk/control drink intake post-exercise (e.g. volume of liquid, taste, etc.); (2) opinion about milk as a functional food for muscle strength/ function; (3) changes in appetite and habitual diet because of milk/control drink intake, and (4) what was liked and disliked about the study (intervention). The post-intervention interview will be completed within 50 minutes.

Statistical methods

As this is a feasibility and acceptability study aimed to inform a larger trial, the focus of data analysis will be descriptive. Using descriptive statistics (percentages, means (SDs)), we will calculate the response rates, the numbers consented and randomised, the retention rate, and the number, length and frequency of interviews and RE sessions. Compliance with the milk and control drink intervention will be calculated as a percentage of actual consumption divided by expected consumption over the 6-week intervention. Recording the number of repetitions for each exercise within each RE session and the weight lifted will allow calculation of several indices of training intensity. Mean and SDs (or equivalent) for questionnaire data and assessments will be reported at screening, baseline and post-intervention interview. Missing data will be recorded and evaluated.

Participants' experiences and views about the study will be assessed with standardised open-ended questions. This data will be analysed using content analysis[52].

For peer review only

Content analysis is a flexible method for analysing text data. Coding categories will be derived directly from the data and themes will be identified supported with relevant quotations of the participant's perspecitves[52].

The sample size in this pilot study is limited to 30 participants and therefore lacks statistical power for quantitative analysis of the secondary outcomes.

ETHICS AND DISSEMINATION

Ethics

The study approval has been granted by the North East–Newcastle and North Tyneside Research Ethic Committee 1 (REC reference number: 18/NE/0265), and Research and Development (R&D) of the Northumbria Healthcare NHS Foundation Trust (Sponsor). The study will be conducted in accordance with the principles of the International Conference for Harmonisation of Good Clinical Practice (European Medicines Agency, 2002). We have amended inclusion criteria for the study, and allowed the inclusion of individuals who have GS or walking speed above the EWGSOP cut-offs[30].

The study is funded by the National Institute for Health Research Newcastle Biomedical Research Centre, Newcastle University. Arla® will provide milk and scientific support related to this nutritional intervention. This study is registered online at https://www.isrctn.com/ISRCTN13398279.

Data monitoring

Throughout the study, the principal investigator (AG) will monitor recruitment, retention and compliance figures with the core research team (AG, CH, LD, TA). The core team will meet regularly to plan and evaluate study's day-to-day activities. Monthly meetings will be organised with the co-investigators (KD, ES, AAS) to update on study management and progress. The core research team and co-investigators will prepare consents, assessments, study protocol, and standard operating procedures for: (1) assessments and

For peer review only

data reporting; (2) data management; (3) adverse events management and reporting, and (4) staff health risk assessment and safety procedures.

Adverse events

This is a low risk study. There is a small chance of transient muscle soreness, gastrointestinal discomfort, metabolic changes, and change in appetite. The chief investigator (TA) is clinically trained to oversee the research process, and the research team is trained in health and safety procedures during data collection. Each participant will be closely monitored and asked about any adverse events occurring at home or in the gym. Any suspected adverse events will be reported to the chief investigator (TA), who will also offer clinical oversight of the study. Any serious adverse events, as evaluated by TA thought to be related to the intervention, will be reported immediately to the study sponsor and relevant ethics committee. Because of the low risk of adverse events, no independent Data Monitoring and Safety Committee will be appointed for this pilot study. The NHS indemnity insurance scheme will apply to cover the potential legal liability cover for harm to participants arising from the research. North Tyneside Council has the public and product liability cover for any potential harm arising from the fitness facility and equipment.

Data management

Data will be collected and managed in accordance with the EU General Data Protection Regulation (2018). At consent, participants will be assigned a unique study ID that will be used to pseudonymise primary research data collected from interviews and intervention. Identifiable data will be stored separately and will be accessible only to members of the research team who have additional research passport checks approved as part of their research role. Pseudonymised paper-based assessments will be double data entered, and all study data will be stored on secure, fire-wall and password protected servers of Newcastle University for 5 years.

For peer review only

Data Statement

Technical appendix, statistical code, and dataset will be available from the AGE Research Group data manager.

Dissemination, and Patient and Public Involvement

The following key outputs will contribute to study dissemination and impact. The results of the study will be reported to the funder (NIHR Newcastle Biomedical Research Centre). The funder, sponsor and industry support (Arla®) will have no role in the study design, conduct, data analysis, results interpretation, or writing. The aim is that at least two peer-reviewed papers will be published in high impact open access journals, and the results will be presented at relevant scientific conferences. A lay summary of the main results will be presented to interested participants at a Public and Patient Involvement event. A flyer featuring the main results of the study and, if desired, an individual report titled 'My muscle function and strength before and after MIlkMAN' will be prepared for all study participants. Reports with abnormal results (blood pressure, BMI, fat mass, MMSE, and GDS) will be send to general practices. Regular updates on the study progress will be reported on a publicly accessible website.

Acknowledgements The authors would like to thank the North East and North Cumbria Clinical Research Network.

Funding This project is funded by the National Institute for Health Research (NIHR) Newcastle Biomedical Research Centre (reference number: BH Ref 173606 / PDB053), Newcastle University and supported by Arla® (in-kind (milk) and scientific advice about nutrition).

Author contribution AG, KD, ES, AAS, TR, LD, and CH developed and refined the study protocol. AG, KD, ES, AAS, and TA were responsible for study conception and design. AG drafted the manuscript. All co-authors revised the manuscript draft. AG was responsible for the analysis plan. All authors were responsible for critical revision and approved the final version of the manuscript.

For peer review only

Funding This project is funded by the National Institute for Health Research (NIHR) Newcastle Biomedical Research Centre (reference number: BH Ref 173606 / PDB053), Newcastle University and supported by Arla® (in-kind milk contribution).

Disclaimer The views expressed are those of the authors and not necessarily those of the NHS or NIHR.

Competing interest This study received 'in-kind' contribution from Arla®.

Ethics approval The North East – Newcastle and North Tyneside Research Ethic Committee 1 (REC reference number: 18/NE/0265).

Provenance and peer review Not commissioned; internally peer reviewed

Open Access This is an open access article distributed in accordance with the Creative Commons Attribution Non Commercial (CC BY-NC 4.0) license, which permits others to distribute, remix, adapt, build upon this work non-commercially, and license their derivative works on different terms, provided that the original work is properly cited, appropriate credit is given, and changes made indicated, and the use is non-commercial. See: http://creaticecommons.org/license/by/4.0/

For peer review only

REFERENCES

1. Office for National Statistics. *Population Estimates for UK, England and Wales, Scotland and Northern Ireland: mid-2016.* https://www.ons.gov.uk/peoplepopulationandcommunity/populationandmigration/populationestimates/bulletins/annualmidyearpopulationestimates/mid2017 (accessed on 10 January 2019).
2. Rowe JW, Kahn RL. Successful aging 2.0: conceptual expansions for the 21st century. *J Gerontol B Psychol Sci Soc Sci* 2015;70:593–6.
3. Drewnowski A, Evans WJ. Nutrition, physical activity, and quality of life in older adults: summary. *J Gerontol A Biol Sci Med Sci* 2001;56:89–94.
4. Cruz-Jentoft AJ, Bahat G, Bauer J, et al. Sarcopenia: revised European consensus on definition and diagnosis. *Age Ageing* 2018 Oct 12. doi: 10.1093/ageing/afy169. [Epub ahead of print]
5. Janssen I, Ross R. Linking age-related changes in skeletal muscle mass and composition with metabolism and disease. *J Nutr Health Aging* 2005;9:408–19.
6. Visser, M.; Schaap, L.A. Consequences of sarcopenia. *Clin Geriatr Med* 2011;27: 387–399.
7. Manrique-Espinoza B, Salinas-Rodríguez, A, et al. Sarcopenia is associated with physical and mental components of health-related quality of life in older adults. *J Am Med Dir Assoc* 2017;18:636.e1–636.e5.
8. Mithal A, Bonjour JP, Boonen, S, et al. Impact of nutrition on muscle mass, strength, and performance in older adults. *Osteoporos Int* 2013;24:1555–66.
9. Marzetti E, Calvani R, Tosato M, et al. Physical activity and exercise as countermeasures to physical frailty and sarcopenia. *Aging Clin Exp Res* 2017;29: 35–42.
10. Cruz-Jentoft AJ, Landi F, Schneider SM, et al. Prevalence of and interventions for sarcopenia in ageing adults: a systematic review. Report of the International Sarcopenia Initiative (EWGSOP and IWGS). *Age Ageing* 2014;43:748–59.
11. Pinedo-Villanueva R, Westbury LD, Syddall HE, et al. Health care costs associated with muscle weakness: A UK population-based estimate. *Calcif Tissue Int* 2018 Sep 22. doi: 10.1007/s00223-018-0478-1. [Epub ahead of print]
12. Volaklis KA, Halle M, Thorand B, et al. Handgrip strength is inversely and independently associated with multimorbidity among older women: Results from the KORA-Age study. *Eur J Intern Med* 2016, 31, 35–40.
13. Tsekoura M, Kastrinis A, Katsoulaki M, Billis E, Gliatis J. Sarcopenia and its impact on quality of life. *Adv Exp Med Biol* 2017;987:213–8.
14. Churchward-Venne TA, Holwerda AM, Phillips SM, et al. What is the optimal amount of protein to support post-exercise skeletal muscle reconditioning in the older adult? *Sports Med* 2016;46:1205–12.
15. Peterson M, Sen A, Gordon P. Influence of resistance exercise on lean body mass in aging adults: a meta-analysis. *Med Sci Sports Exerc* 2011;43:249–58.
16. Pennings B, Koopman R, Beelen M, et al. Exercising before protein intake allows for greater use of dietary protein–derived amino acids for de novo muscle protein synthesis in both young and elderly men. *Am J Clin Nutr* 2011;93:322–31.
17. Witard OC, Jackman SR, Breen L, et al. Myofibrillar muscle protein synthesis rates subsequent to a meal in response to increasing doses of whey protein at rest and after resistance exercise. *Am J Clin Nutr* 2014;99:86–95.

For peer review only

18. Moore DR, Churchward-Venne TA, Witard O, et al. Protein ingestion to stimulate myofibrillar protein synthesis requires greater relative protein intakes in healthy older versus younger men. *J Gerontol A Biol Sci Med Sci* 2015;70:57–62.
19. Tieland M, Dirks ML, van der Zwaluw N, et al. Protein supplementation increases muscle mass gain during prolonged resistance-type exercise training in frail elderly people: a randomized, double-blind, placebo-controlled trial. *J Am Med Dir Assoc* 2012;13:713–9.
20. Millward DJ. Nutrition and sarcopenia: evidence for an interaction. *Proc Nutr Soc* 2012;71:566–75.
21. Appleton KM. Barriers to and facilitators of the consumption of animal-based protein-rich foods in older adults. *Nutrients* 2016;8:187.
22. Pereira PC. Milk nutritional composition and its role in human health. *Nutrition* 2014;30:619–27.
23. Naclerio F, Larumbe-Zabala E. Effects of whey protein alone or as part of a multi-ingredient formulation on strength, fat-free mass, or lean body mass in resistance-trained individuals: a meta-analysis. *Sports Med* 2016;46:125–37.
24. Tang JE, Moore DR, Kujbida GW, Tarnopolsky MA, Phillips SM. Ingestion of whey hydrolysate, casein, or soy protein isolate: effects on mixed muscle protein synthesis at rest and following resistance exercise in young men. *J Appl Physiol (1985)* 2009;107:987–92.
25. Yang Y, Breen L, Burd NA, et al. Resistance exercise enhances myofibrillar protein synthesis with graded intakes of whey protein in older men. *Br J Nutr* 2012;108:1780–8.
26. Elliot TA, Cree MG, Sanford AP, et al. Milk ingestion stimulates net muscle protein synthesis following resistance exercise. *Med Sci Sports Exerc* 2006;38:667–74.
27. Rankin P, Stevenson EJ, Cockburn E. The effect of milk on the attenuation of exercise-induced muscle damage in males and females. *Eur J Appl Physiol* 2015;115:1245–61.
28. Rankin P, Landy A, Stevenson E, et al. Milk: an effective recovery drink for female athletes. *Nutrients* 2018;10(2).
29. Malmstrom TK, Morley JE. SARC-F: a simple questionnaire to rapidly diagnose sarcopenia. *J Am Med Dir Assoc* 2013;14:531–2.
30. Cruz-Jentoft AJ, Baeyens JP, Bauer JM, et al.; European Working Group on Sarcopenia in Older People. Sarcopenia: European consensus on definition and diagnosis: Report of the European Working Group on Sarcopenia in Older People. *Age Ageing* 2010;39:412–23.
31. Guralnik JM, Simonsick EM, Ferrucci L, et al. A short physical performance battery assessing lower extremity function: association with self-reported disability and prediction of mortality and nursing home admission. *J Gerontol* 1994;49:M85–94.
32. Moon JR, Stout JR, Smith-Ryan AE, et al. Tracking fat-free mass changes in elderly men and women using single-frequency bioimpedance and dual-energy X-ray absorptiometry: a four-compartment model comparison. *Eur J Clin Nutr* 2013;67 Suppl 1:S40–6.
33. Roberts HC, Denison HJ, Martin HJ, et al. A review of the measurement of grip strength in clinical and epidemiological studies: towards a standardised approach. *Age Ageing* 2011;40:423–9.

For peer review only

34. Gandek B, Ware JE, Aaronson NK, et al. Cross-validation of item selection and scoring for the SF-12 Health Survey in nine countries: results from the IQOLA Project. International Quality of Life Assessment. *J Clin Epidemiol* 1998;51:1171–8.
35. Wade DT, Collin C. The Barthel ADL Index: a standard measure of physical disability? *Int Disabil Stud* 1988;10:64–7.
36. Saghaei, M, Saghaei, S. Implementation of an open-source customizable minimization program for allocation of patients to parallel groups in clinical trials. *J Biomed Sci Eng* 2011;4:734–9.
37. Chandola T, Jenkinson C. The new UK National Statistics Socio-Economic Classification (NS-SEC); investigating social class differences in self-reported health status. *J Public Health Med* 2000;22:182–90.
38. Smith T, Noble M, Noble S, et al. *The English Indices of Deprivation 2015*. London: Department of Communities and Local Government; 2015. https://assets.publishing.service.gov.uk/government/uploads/system/uploads/attachment_data/file/464485/English_Indices_of_Deprivation_2015_-_Technical-Report.pdf. Accessed April 12, 2018.
39. Brown LM, Schinka JA. Development and initial validation of a 15-item informant version of the Geriatric Depression Scale. *Int J Geriatr Psychiatry* 2005;20:911–8.
40. Simpson E, Bradley J, Poliakov I, et al. Iterative development of an online dietary recall tool: INTAKE24. *Nutrients* 2017;9(2).
41. Wilson MM, Thomas DR, Rubenstein LZ, et al. Appetite assessment: simple appetite questionnaire predicts weight loss in community-dwelling adults and nursing home residents. *Am J Clin Nutr* 2005;82:1074–81.
42. Collerton J, Barrass K, Bond J, et al. The Newcastle 85+ study: biological, clinical and psychological factors associated with healthy ageing: study protocol. *BMC Geriatr* 2007;7:14.
43. Robinson MM, Dasari S, Konopka AR, et al. Enhanced protein translation underlies improved metabolic and physical adaptations to different exercise training modes in young and old humans. *Cell Metab* 2017;25:581–92.
44. Peterson MD, Gordon PM. Resistance exercise for the aging adult: clinical implications and prescription guidelines. *Am J Med* 2011;124:194–8.
45. Borde R, Hortobágyi T, Granacher U. Dose-response relationships of resistance training in healthy old adults: a systematic review and meta-analysis. *Sports Med* 2015;45:1693–720.
46. Brzycki, M. Strength testing—predicting a one-rep max from reps-to-fatigue. *J Phys Educ Recreat Dance* 1993;64:88–90.
47. Whelton PK, Carey RM, Aronow WS, et al. 2017 ACC/AHA/AAPA/ABC/ACPM/AGS/APhA/ASH/ASPC/NMA/PCNA Guideline for the Prevention, Detection, Evaluation, and Management of High Blood Pressure in Adults: Executive Summary: A Report of the American College of Cardiology/American Heart Association Task Force on Clinical Practice Guidelines. *Circulation* 2018;138:e426–83.
48. Rezk CC, Marrache RC, Tinucci T, et al. Post-resistance exercise hypotension, hemodynamics, and heart rate variability: influence of exercise intensity. *Eur J Appl Physiol* 2006;98:105–12.
49. Huggett DL, Elliott ID, Overend TJ, et al. Comparison of heart-rate and blood-pressure increases during isokinetic eccentric versus isometric exercise in older adults. *J Aging Phys Act* 2004;12:157–69.

For peer review only

50. Borg E, Borg G. A comparison of AME and CR100 for scaling perceived exertion. *Acta Psychol (Amst)* 2002;109:157–5.
51. The Food Standards Agency (2002). *McCance and Widdowson's the Composition of Foods Integral Dataset* (6th Summary Edition). http://webarchive.nationalarchives.gov.uk. Accessed in: August 20, 2017
52. Hsieh HF, Shannon SE. Three approaches to qualitative content analysis. *Qual Health Res* 2005;15:1277–88.

For peer review only

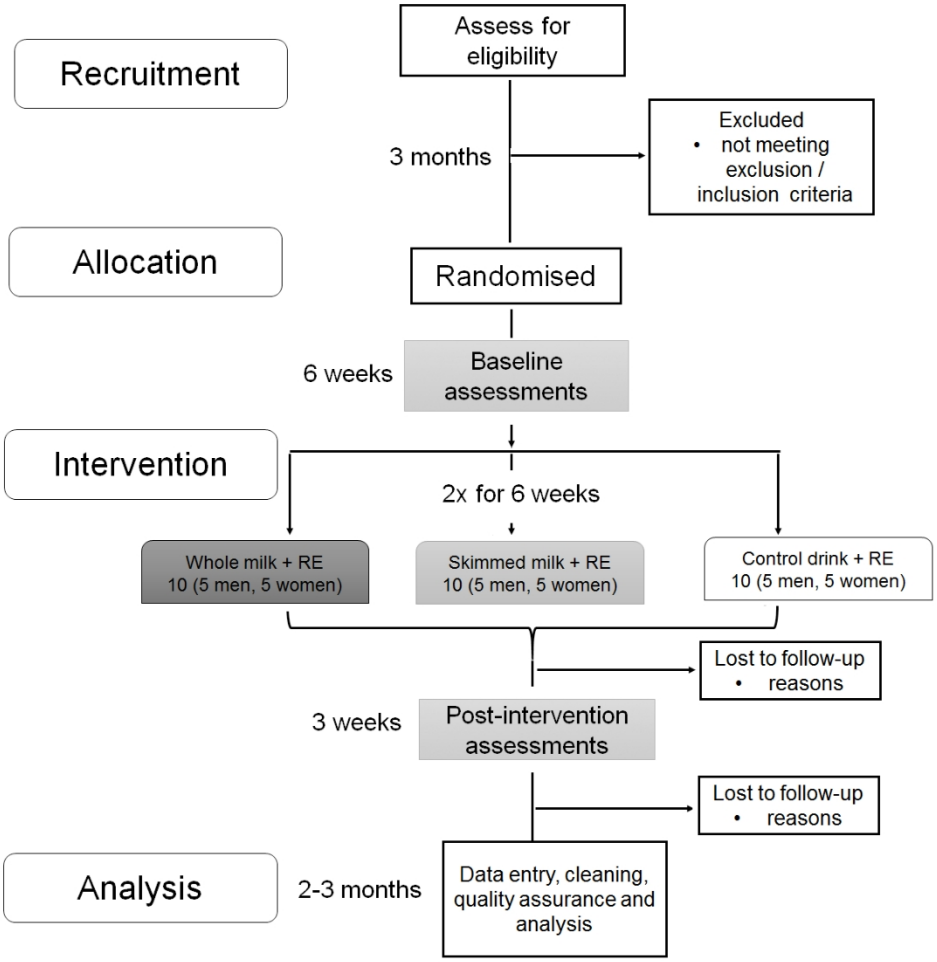

Figure 1. Study flow chart.

109x111mm (300 x 300 DPI)

SUPPLEMENTARY MATERIAL

Appendix 1

THE SARC-F QUESTIONNAIRE[29]

Strength: How much difficulty do you have in lifting and carrying 10 pounds / a bag of shopping?
None: 0
Some: 1
A lot or unable: 2

Assistance in walking: How much difficulty do you have walking across a room?
None: 0
Some: 1
A lot, use aids, or unable: 2

Rise from a chair: How much difficulty do you have transferring from a chair or bed?
None: 0
Some: 1
A lot or unable without help: 2

Climb stairs: How much difficulty do you have climbing a flight of 10 stairs?
None: 0
Some: 1
A lot or unable: 2

Falls: How many times have you fallen in the past year?
None: 0
1 to 3 falls: 1
4 or more falls: 2

For peer review only

Appendix 2

R100® scale[50]

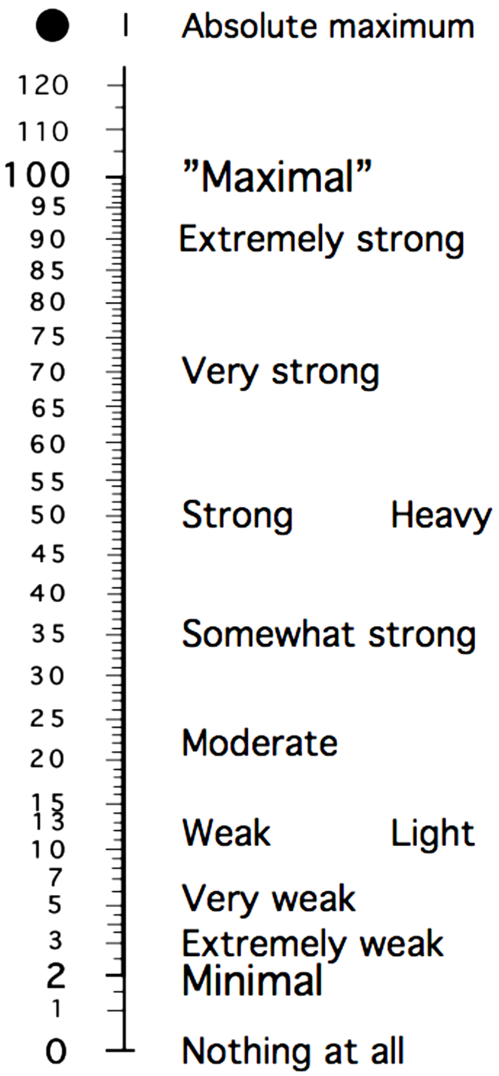

Instructions for participants (explained to participants by exercise physiologist)
Assessment ~10 minutes after exercise

- Using the scale, we would like you to rate your perceptions of EFFORT (RPE), that is, how difficult the session felt to you.
- Your perception of EFFORT should be a conscious awareness of how hard (or easy) the whole session was. It should not be influenced your feelings of fatigue, pain or discomfort (try separating these from effort as best you can).
- You will be asked to rate your perceptions of overall effort (the whole session after the warm-up), and then separate scores for upper-body muscle effort (arm muscles) and lower-body muscle effort (leg muscles).

review only

Appendix 3

Visual analogue scale for muscle soreness

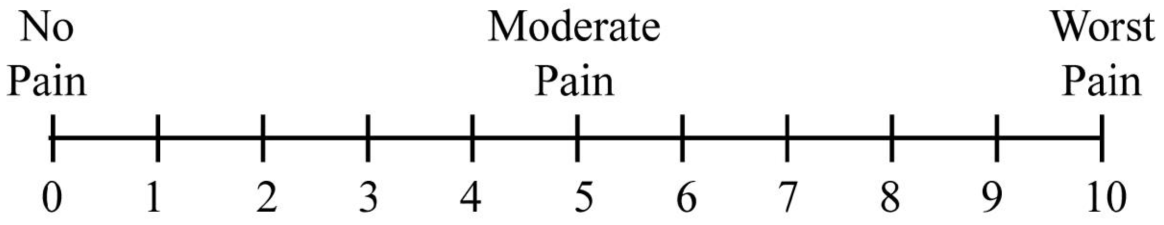

0 = no pain, 1-3 = mild pain, 4-6 = moderate pain; 7-10 = severe

Instructions for participants (explained to participants by a researcher)

- This is a Visual Analogue Scale. The scale describes the intensity of your muscle soreness, 0 meaning no pain at all, 1 to 3 meaning mild pain, 4-6 moderate pain, and 7-10 the worst pain.
- Assessment ~45 minutes after exercise: Which number on the scale describes the best your muscle soreness in your arms and legs?
- *Assessment in the evening over the telephone (6-7 hours after exercise)*: A researcher will call you in the evening after each visit to the gym to ask you again about your muscle soreness in your arms and legs. Please use this visual analogue scale to rate your muscle soreness in your arms and legs.

For peer review only

BMJ Open

Milk and resistance exercise intervention to improve muscle function in community-dwelling older adults at risk of sarcopenia (MIlkMAN): protocol for a pilot study

Journal:	*BMJ Open*
Manuscript ID	bmjopen-2019-031048.R1
Article Type:	Protocol
Date Submitted by the Author:	15-Jul-2019
Complete List of Authors:	Granic, Antoneta; Newcastle University, Institute of Neuroscience<br>Hurst , Christopher; Newcastle University, Institute of Neuroscience<br>Dismore , Lorelle ; Newcastle University, Institute of Neuroscience<br>Davies , Karen ; Newcastle University, Institute of Neuroscience<br>Stevenson , Emma ; Newcastle University, Institute of Cellular Medicine<br>Sayer, Avan; Newcastle Upon Tyne Hospitals NHS Foundation Trust, NIHR Biomedical Research Centre; Newcastle University<br>Aspray , Terry ; Newcastle University, Institute of Cellular Medicine
**Primary Subject Heading**:	Geriatric medicine
Secondary Subject Heading:	Nutrition and metabolism, Patient-centred medicine
Keywords:	pilot study, older adults, sarcopenia, physical function, whole milk, resistance exercise

SCHOLARONE™
Manuscripts

Milk and resistance exercise intervention to improve muscle function in community-dwelling older adults at risk of sarcopenia (MIlkMAN): protocol for a pilot study

Antoneta Granic[1-3*], Christopher Hurst[1,2†], Lorelle Dismore[1,2†], Karen Davies[1-3], Emma Stevenson[4,5], Avan A Sayer[1-3], Terry Aspray[1,2,4, 6]

[1]AGE Research Group, Institute of Neuroscience, Newcastle University, Newcastle upon Tyne, United Kingdom

[2]NIHR Newcastle Biomedical Research Centre, Newcastle upon Tyne Hospitals NHS Foundation Trust and Newcastle University, Newcastle upon Tyne, United Kingdom

[3]Newcastle University Institute for Ageing, Newcastle upon Tyne, United Kingdom

[4]Institute of Cellular Medicine, Newcastle University, Newcastle upon Tyne, United Kingdom

[5]Human Nutrition Research Centre, Newcastle University, Newcastle upon Tyne, United Kingdom

[6]Newcastle upon Tyne Hospitals NHS Foundation Trust, Musculoskeletal Unit, Freeman Hospital, Newcastle upon Tyne, United Kingdom

[†]equal contribution

For peer review only

*correspondence:

E-mail: antoneta.granic@newcastle.ac.uk

Phone: +44 (0) 1912081112

Biomedical Research Building, 1st Floor

Campus for Ageing and Vitality

Newcastle University

Newcastle upon Tyne, NE4 5PL

United Kingdom

Word count: 4,782

For peer review only

ABSTRACT

Introduction: Sarcopenia is a progressive muscle disorder characterised by decline in skeletal muscle mass, strength and function leading to adverse health outcomes, including falls, frailty, poor quality of life, and death. It occurs more commonly in older people and can be accelerated by poor diet and low physical activity. Intervention studies incorporating higher dietary protein intakes or protein supplementation combined with resistance exercise (RE) have been shown to limit muscle function decline. However, less is known about the role of whole foods in reducing the risk of sarcopenia. Milk is a source of high-quality nutrients, which may be beneficial for skeletal muscle. This pilot study examines the feasibility and acceptability of milk consumption with RE to improve muscle function in community-dwelling older adults at risk of sarcopenia.

Methods and Analysis: 30 older adults aged ≥65 years will be randomly allocated to three groups: 'whole milk + RE', 'skimmed milk + RE' or 'control drink + RE'. Assessments will take place in participants' homes, including screening (milk allergies, grip strength, walking speed), baseline and post-intervention health and function. All participants will undertake a structured RE intervention twice/week for 6 weeks at a local gym, followed by the consumption of 500ml of whole or skimmed milk (each ~20 g of protein) or an isocaloric control drink and another 500ml at home. Participants' views about the study will be assessed using standardised open-ended questions. The primary outcomes include feasibility and acceptability of the intervention with recruitment, retention, and intervention response rates. Analyses will include descriptive statistics, exploration of qualitative themes and intervention fidelity.

Ethics and dissemination: The North East–Newcastle and North Tyneside Research Ethic Committee 1 (18/NE/0265) approved the study. Outputs include pilot data to support funding applications; public involvement events; presentation at conferences, and peer-reviewed publication.

Trail registration number: ISRCTN13398279; Pre-results.

For peer review only

Keywords: pilot study, older adults, physical function, muscle strength, sarcopenia, whole milk, resistance exercise, dietary protein, quality of life

Strengths and limitations of this study

- To our knowledge, this is the first pilot study examining the feasibility and acceptability of the whole versus skimmed milk with resistance exercise (RE) intervention in community-dwelling older adults living in the UK.
- The intervention is conducted in a local gym that is easily accessible to older adults who will benefit from the familiarisation with RE programme conducted in the community to foster continuous engagement.
- Post-intervention interview will allow for the collection of qualitative evidence to support planned future trial, including better understanding of the barriers and facilitators of community-based intervention.
- Because this an evaluation of a pilot implementation, the sample size is not based on statistical power.
- Although we do not anticipate any definite results in exploring differences between intervention groups, the results will be used to aid power calculations for planned future substantive research.

only

INTRODUCTION

The UK population is ageing rapidly; the number of adults aged ⩾65 increased by 17.3% in the last decade, and in mid-2017 was estimated to account for 18.2% of the total population of 66 million[1]. Understanding factors associated with healthy ageing[2] such as diet and physical activity[3] for optimising health and wellbeing of an ageing population is essential for the development of effective interventions.

Sarcopenia is a progressive, generalised muscle disorder characterised by decline in skeletal muscle mass, strength and function[4], which leads to an increased risk of falls, frailty, disability, low health-related quality of life (QoL) and death[5-8]. It occurs more commonly in older people and can be accelerated by poor diet and low physical activity[8,9]. The prevalence of sarcopenia increases with advancing age—and although dependent on the algorithm used to define sarcopenia[10]—it reaches more than 20% in men and women aged ≥85 years[11], resulting in an estimated excess of health care cost of £2.5 billion/year in the UK[12]. This emphasises the need for sustainable preventive measures aimed to preserve and optimise muscle health and function in a rapidly ageing population before the onset of difficulties leading to or exacerbating the risk of sarcopenia.

Protein intake and exercise for healthy muscle ageing

Loss of muscle mass and strength can be accelerated by poor diet, low levels of physical activity and the presence of long-term conditions[8,9,13], leading to diminished QoL[7,14]. Adequate intake of dietary protein and resistance exercise (RE) are recognised as effective interventions to promote skeletal muscle health and reduction of physical decline[15,16]. Specifically, intervention studies that examined a combined effect of protein supplements, comprising essential amino acids (EAA) and RE to stimulate muscle protein synthesis (MPS) have observed an increase in total muscle protein within 3-5 hours following exercise in both young and older adults[17]. Compared with young adults, older adults experience a blunted response after protein ingestion to stimulate MPS (anabolic resistance), especially in

For peer review only

response to lower amounts of protein or EAA of <20g or <10g, respectively. Other studies have shown that greater amounts of protein supplementation and intermittent feeding in combination with repeated bouts of RE resulted in increased muscle mass in older adults, even in those diagnosed with frailty and sarcopenia[18-20].

However, there is limited research on the role of whole foods rich in protein (e.g. milk and dairy products, fish, and meats) in maintaining skeletal muscle mass, strength and function in older adults at risk of sarcopenia. Regular consumption of high-quality, nutrient-dense foods, high in macro- and micronutrients relevant for muscle[21] within a varied diet may provide a platform for developing strategies for maintenance of muscle health and function in later life that do not include supplements and medical products, and may be easier adopted as a behavioural change in older adults[22].

Milk for muscle health: current evidence and why this pilot is needed

Cow's milk is an example of a whole food with the potential to ameliorate loss of skeletal muscle mass, strength and function. Whole milk (3.6% fat) is a source of high-quality proteins (whey and caseins), minerals (e.g. calcium, phosphorus, magnesium), vitamins (e.g. A, B, D, and E), carbohydrates, bioactive lipids and fatty acids (mono- and polyunsaturated, and saturated fatty acids)[23]. Whey protein is considered superior to other protein sources for MPS after exercise in younger and older adults because of its greater bioavailability and solubility, and higher content of the branched-chain amino acids, including leucine[24-26]. Furthermore, the concurrent intake of milk fats with protein in whole milk has been shown to increase the use of EAA for MPS after exercise in young men compared with skimmed milk (0.3% fat)[27], suggesting additional benefits of milk lipids for muscle. Other benefits of milk containing fat include reduction in exercise-related muscle damage, soreness, and decline in muscle performance in young adults and athletes[28,29] compared with energy–matched (isocaloric) carbohydrate drink. However, little is known about the effect of milk and protein-fat ratio in milk on muscle in older adults, particularly the impact on

For peer review only

muscle function of varying milk fat contents (whole versus skimmed) providing >20g protein/day after exercise.

We hypothesised that whole milk (3.6% fat), providing >20g of protein and the same amount of energy as fat and protein-free carbohydrate drink, after structured exercise conducted in the community may be a feasible and acceptable intervention for maintaining skeletal muscle mass, strength and function in older adults at risk of sarcopenia.

Study aims

The primary aims are:

(1) To examine the feasibility and acceptability of whole (3.6% fat) or skimmed milk (0.3% fat) in combination with RE as an intervention in community-dwelling older adults aged ≥65 at risk of sarcopenia. This aim will answer the following questions: Is an intervention of 2 × 500ml milk + RE twice/week for 6 weeks (a) feasible and (b) acceptable to older adults?

(2) To provide essential data for planned future substantive research.

The secondary aim of the study will be to explore whether consumption of whole or skimmed milk + RE has an influence on physical performance, muscle mass, strength and self-reported QoL in older adults at risk of sarcopenia.

For peer review only

METHODS AND ANALYSIS

Study design

This is a pilot study with a parallel group design involving 30 participants (aiming for 15 men and 15 women) aged ≥65 who will be randomised into three intervention groups: (group 1) 'whole milk + RE'; (group 2) 'skimmed milk + RE', and (group 3) 'control drink' + RE. Data will be collected from: (1) health and functioning assessments (screening, baseline and post-intervention interview); (2) the nutrition + exercise intervention over 6 weeks, and (3) participants' feedback about the study.

Exclusion and inclusion criteria

The study will include older adults who are registered patients with General Practitioner (GP) practices within the National Institute for Health Research (NIHR) North East and North Cumbria Clinical Research Network (CRN), United Kingdom. Table 1 lists the inclusion and exclusion criteria that are applied to patient database searches, performed in GP practices and screening interviews conducted by the research team.

Table 1. Inclusion and exclusion criteria for the MIlkMAN: pilot

Criteria	Patient database searches	Screening interview
Inclusion	aged 65 and over	
	live in the community	
Exclusion	diabetes mellitus type 1 or type 2	lacks capacity to consent to participate
	chronic kidney disease stage 4 or 5 (estimated glomerular filtration rate <30ml / min /1.73m^2)	lactose intolerance
	liver function impairment (AST >2.5 times upper limit of normal range within the last 6 months)	dislikes milk or cranberry juice (control drink)
	chronic lung disease requiring maintenance steroid therapy (e.g. COPD, severe asthma)	participated in a structured RE training and gym programme in the last month
	end-stage terminal illness	dislikes gym exercise with equipment
	cardiac pacemaker or severe heart failure or other significant heart disease	unintentional weight loss ⩾5kg in the last 3 months
	uncontrolled hypertension (>160/100 mmHg) and uncontrolled hypotension (<100 mmHg systolic) within last 6 months	unable to understand instructions for muscle strength and function assessments in English or unwilling to participate in protocol when explained
	hip or knee replacement	an individual who the research team (exercise physiologist) evaluates as not suitable for the intervention because of safety reasons
	impaired mobility (unable to walk without an aid including wheelchair	
	current prescription of warfarin (potential interference with control drink)	
	BMI ⩾30kg/m2	
	an individual who the GP feels it is inappropriate for the research team to	

For peer review only

	approach for safety reasons: any medical and physical conditions that preclude safe participation in a RE programme (long-term conditions likely to lead impaired function over 6 months)

AST, Aspartate Aminotransferase; BMI, body mass index; COPD, Chronic Obstructive Pulmonary Disease; GP, general practitioner.

Study population and recruitment

Primary care recruitment will be carried out with the assistance of North East and North Cumbria CRN, England, which provides support with access to general practices and their patients. Two practices in North Tyneside Clinical Commissioning Group were identified for feasibility using exclusion/inclusion criteria (Table 1, left column) and provided feedback during the application for funding in 2018.

Recruitment will be organised in two stages: pre-screening (GP practices) and screening (research team). At the pre-screening stage, practices will identify potential participants from their patient database using exclusion/ inclusion criteria (Table 1, left column), and then mail out recruitment packs, containing detailed information about the study with a reply slip. Interested individuals will be interviewed over the telephone by a researcher using a 5-item SARC-F questionnaire (Appendix 1)[30] to assess any difficulties with day-to-day activities (lifting and carrying 10 pounds, walking, rising from a chair and climbing stairs), and number of falls in the past year. Those evaluated by the research team to have no major difficulties that would preclude safe participation in the exercise programme (e.g. unable to walk across a room), will be visited in their own home for a screening interview to obtain written informed consent, and to evaluate inclusion/exclusion criteria not screened through GPs (Table 1, right column).

Those who meet the criteria will be assessed further for muscle strength (grip strength, GS) and function (walking speed) based on the following cut-offs: <20 kg (women), and <30 kg (men)[31] for low GS; and <0.8 m/s or ⩾5 s over 4 m distance[31] for low walking speed. GS measurements (high or low) at the screening interview will be used for

For peer review only

minimisation along with sex to allow equal distribution of those with muscle strength weakness across the intervention groups. However, the target number of those with 'low' GS will not be established a priori. Therefore, the study will recruit older adults with some deficits in muscle health and those without for whom it is determined to be safe to participate in the study (primary aims), and hypothesised to benefit from the intervention regardless of the deficits (secondary aims).

Study outcomes

Table 2 lists the primary and secondary study outcomes and when they are completed.

Table 2. Study outcome measures

Measure	Screening	Baseline	Post-intervention
Primary			
Feasibility and acceptability of intervention in a local gym setting			×
Applicability			×
Dosage and duration of intervention			×
Compliance		×	×
Attrition		×	×
Adverse health effects			×
Response rates to questionnaires, assessments, and intervention	×	×	×
Secondary			
Short Physical Performance Battery[32] (balance, 4m-gait speed, 5-chair stands)		×	×
Muscle mass[33]		×	×
Grip strengh[34]	×	×	×
SF-12 Health Survey[35]		×	×
Barthel Index[36]		×	×

The secondary outcome measures will explore differences in physical performance measures between the groups, pre and post-intervention. GS (muscle strength)[34] will be expressed as the maximum reading of 6 trials of both hands using a Jamar hand-held 5030J1 dynamometer. Body composition (muscle mass) will be measured using Bioelectric Impedance Analysis[33] (BIA; Tanita MC-780MA Body Composition Analyzer). Self-reported QoL will be measured using SF-12[35], and activities of daily living with Barthel Index[36].

For peer review only

Randomisation

A researcher will allocate thirty participants to one of the three interventions to ensure balanced allocation of participants between the groups based on sex and muscle strength (GS at screening assessment) using a free, open-source minimisation software (MiniPy 0.3, http://minimpy.sourceforge.net)[37]. The software features elements of randomness in the minimisation algorithm by allocating the first participant randomly into one of the interventions, and assigning the subsequent participants on hypothetical stepwise allocation to every group and computation of the imbalance score corresponding to each allocation. The imbalance scores are compared and participants allocated to the group corresponding to the least imbalance score (preferred group).

The sample size for the pilot is not based on statistical power but guided by the consideration to fulfil the primary aims of the study (e.g. provide guidelines for the larger trial) and practical feasibility[38].

Consent

Written informed consent will be obtained by a researcher visiting the participants during the home-based screening assessment prior to randomisation. Capacity to consent will be assessed using an established consent pathway. Throughout the active research phase (i.e. from baseline to post-intervention assessment), the notion of process consent will be implemented, requiring an ongoing exchange of information about the study and confirming the participants willingness to proceed, ensuring that participants are free to reconsider and withdraw from the study at any time. If a participant loses capacity during the research process, he/she will be withdrawn from the study.

Flow diagram of the study

A flow diagram of the study protocol with timelines is outlined in Figure 1. The recruitment (assessment of eligibility) up to allocation (randomisation into 3 groups) will be

For peer review only

finalised within 3 months, followed by baseline assessments for health and functioning in participants' homes, and a 6-week intervention in a local gym.

>>Insert Figure 1<<

Figure 1. Study flow chart.

Home-based post-intervention assessments, including participants' feedback about the study, will be conducted the week following completion of the intervention, and finalised within 3 weeks. Data analysis will be completed after active data collection (from randomisation to post-intervention assessment). Data collection for each participant will span approximately 10 weeks: (1) week 1: consent and home-based screening assessment; (2) week 2: home-based baseline interview; (3) week 3 to week 8: 6-week intervention twice/week (12 visits at a local gym/sport centre); (4) week 10: post-intervention home-based interview. Except for the intervention (6 consecutive weeks), this time scale can be adjusted to participants' individual needs with a maximum 3 weeks gap between baseline assessment and the first week of intervention, and a maximum 3 weeks gap between the last week of intervention and post-intervention assessment.

Data collection

To determine the feasibility and acceptability of the study, the following data will be collected and analysed. The number of individuals approached; the reasons for not opting to take a part in the study (reported with permission); the recruitment and retention rates; the reasons for attrition; the completion of objective assessments and questionnaires; the number of RE sessions completed, and compliance with the milk/control drink intake. Other health and functioning data will be collected at the home-based screening and baseline interview, during the intervention (at the gym), and at the home-based post-intervention interview. Participants' attitudes and opinions about the study will be collected at the post-intervention interview using a combination of multiple-response and standardised open-ended questions.

For peer review only

Screening interview

Once potential participants have been identified by the GP practices, having expressed an interest in participation and being interviewed over the phone by a researcher, a mutually convenient appointment will be arranged for a screening visit at the participant's home. Participants will be screening for other exclusion/ inclusion criteria not assessed at by the GP practices (Table 1, right column), and to establish participants' muscle strength (GS) and functioning status (walking speed) based on the established cut-offs[31]. Informed consent will be obtained before any assessment is undertaken. Eligible individuals will be informed about the study procedure and their journey through the study (from randomisation to post-intervention assessment).

Baseline interview

Table 3 lists domains and assessments for the baseline interview with times needed to administer (in minutes). The detailed health and functioning profile will involve minimal risk and inconvenience to participants, and it will be conducted within 70 minutes (including breaks and excluding assessments done at the gym).

Table 3. Domains and assessments at baseline

**Domain and assessment**	**Time to administer (min)**
Sociodemographic profile	total: 6
age	
sex	
marital status	
education	
social class (NS-SEC)[39]	
deprivation (Multiple Index of Deprivation)[40]	
General health	total: 54
SF-12 Health Survey[35]	4
self-reported diseases diagnosed by a doctor	2
list of medication (prescribed and over-the-counter)	2
Mini Mental State Examination	10
Geriatric Depression Scale (15-item version)[41]	7
Barthel Index (Activities of Daily Living)[36]	3
blood pressure (systolic and diastolic)*	5
Intake24: 24-hr dietary recall[42]* (https://intake24.co.uk/)	20
appetite (a 4-item Simplified Nutritional Appetite Questionnaire)[43]*	1
Lifestyle	total: 5
self-reported physical activity[44]	3
smoking status	1

For peer review only

alcohol intake	1
Anthropometry	total: 14
demi-span	2
waist and hip circumference	3
calf circumference	2
muscle mass (body composition by BIA)[33]*	7
Physical functioning	total: 24
Short Physical Performance Battery (SPPB)[32]	10
balance (a side-by-side tandem; semi-tandem; tandem)	3
4-m gait speed	3
5-chair stands	4
maximum grip strength (measured 3 times in each arm)	4

*Assessments done at the intervention site (gym) before and after each RE session (blood pressure), and before the first RE session (diet, appetite, body composition).

Intervention

Resistance exercise (RE)

All participants will perform two RE sessions per week for 6 weeks at a community leisure centre (The Parks, North Tyneside Council, North Shields, UK) that is easily accessible and close to their residence. For each RE session a time slot of ~45-60 min in duration will be allocated, with a minimum of 48 hours between sessions. The sessions will be completed in groups of 2-4 participants under the supervision of an experienced exercise physiologist (CH). Exercise intensity, volume, frequency and duration have been determined based on recent literature[45-47] and the American College of Sports Medicine (ACSM) recommendations for older adults[46]. With the exception of the structured exercise sessions and the nutritional intervention prescribed, participants will be asked to maintain their usual diet, level of physical activity and lifestyle throughout the duration of the intervention period.

During the first RE session participants will be familiarised with the exercises (leg press, leg curl, seated row, chest press) as well as the equipment to be used throughout the intervention with correct technique demonstrated and extensively described. Following this, participants' one repetition maximum (1RM) will be estimated for all four exercises using a previously established equation[48].

Following the initial RE session, each remaining session will begin with a 5-minute warm-up performed at progressive intensity using either a cycle ergometer or treadmill.

For peer review only

Participants will then complete 2-4 sets of 8-12 repetitions at a workload of 70-79%1RM[45,46] for all four of the exercises listed above. Each session will conclude with a short cool-down period of low intensity aerobic exercise, and (except the initial session) will be completed within 30 minutes.

In an attempt to promote participants' engagement with RE, each will receive a training log with diagrams and short instructions with space to record the details of the exercise completed. Participants' gym attendance, sets and repetitions completed, and weight lifted will be recorded following each RE session allowing for the calculation of measures of training load (e.g. volume load [number of sets × number of repetitions × weight lifted]). In addition to measures of external training load, resistance training intensity will be monitored using participant ratings of perceived exertion. Using the CR100® scale[49] (Appendix 2), participants will provide an overall session rating of perceived exertion (sRPE) as well as differential ratings of perceived exertion for upper-body muscle exertion (RPE-U) and lower-body muscle exertion (RPE-L) approximately 10 minutes after the completion of each RE session[50]. Each participant must complete at least 10 sessions (out of 12) to be considered compliant with the exercise programme.

Blood pressure and heart rate will be measured pre and post each RE session in each participant and compared to the guidelines provided by the American College of Cardiology/American Heart Association Task Force[51] and existing literature[52,53]. Muscle soreness will be assessed using a simple visual analogue scale (Appendix 3) at ~40-45 minutes and at ~6-7 hours after each RE session.

Nutritional intervention

On average, 500ml milk contains ~20g of protein needed to stimulate MPS above stimulation provided by RE[17,18]. Whole cow milk (nutritional estimates of 22 UK samples during winter and summer) provides 66 kcal/100g of energy[54]. Arla Cravendale® whole milk contains 3.6g fat, 3.4g protein, and 4.7g of carbohydrates per 100g of milk. Arla

For peer review only

Cravendale® skimmed milk contains 0.3g fat, 3.6g protein, and 4.9g of carbohydrate per 100g of milk. The energy content of the control drink (cranberry juice; Ocean Spray Classic®; 23/kcal/100g of energy) will be balanced to match whole milk energy content and supplemented with maltodextrin (4kcal/g; www.myprotein.com) on the day of intervention. Both milk and juice will be provided in packs of 1L, bought fresh on a weekly basis through a local retailer and kept in a locked refrigerator at the Campus for Ageing and Vitality, Newcastle University.

The milk/control drink will be consumed as a bolus intake of 500ml under the supervision of a researcher immediately after exercise during the recovery period, aiming for complete consumption within ~45 minutes prior to leaving the centre. The second dose of 500ml will be consumed at the participants' home over the next 4-5 hours as a part of their usual diet with other foods. Participants' compliance with consumption of the milk/control drink will be checked in the evening (~6-7 hours post-exercise). Each participant will be provided with a plastic measuring jug (500ml) to measure their consumption at home and to report it back to a researcher over the telephone.

Post-intervention interview

Table 3 lists the domains and assessments that will be repeated at the post-intervention interview. Briefly, a home visit will be arranged with each participant after the 6-week intervention to assess his/her general health and physical functioning, including SPPB, muscle mass (body composition), muscle strength (GS), SF-12, Barthel Index, diet and appetite. Additionally, participants' feedback will be collected at the end of the post-intervention interview using a combination of structured multiple-response and standardised open-ended questions. The following themes will be explored: (1) attitudes and barriers to consuming of 2 × 500ml milk/control drink intake post-exercise (e.g. volume of liquid, taste, etc.); (2) opinion about milk as a functional food for muscle strength/ function; (3) changes in appetite and habitual diet because of milk/control drink intake, and (4) what was liked and disliked about the study (intervention), including motivations and barriers to continue

For peer review only

engagement in a local gym. The post-intervention interview will be completed within 50 minutes.

Statistical methods

As this is a feasibility and acceptability study aimed to inform a larger trial, the focus of data analysis will be descriptive. Using descriptive statistics (percentages, means (SDs)), we will calculate the response rates, the numbers consented and randomised, the retention rate, and the number, length and frequency of interviews and RE sessions. Compliance with the milk and control drink intervention will be calculated as a percentage of actual consumption divided by expected consumption over the 6-week intervention. Recording the number of repetitions for each exercise within each RE session and the weight lifted will allow calculation of several indices of training intensity. Mean and SDs (or equivalent) for questionnaire data and assessments will be reported at screening, baseline and post-intervention interview. Missing data will be recorded and evaluated.

Participants' experiences and views about the study will be assessed with standardised open-ended questions. This data will be analysed using content analysis[55]. Content analysis is a flexible method for analysing text data. Coding categories will be derived directly from the data and themes will be identified supported with relevant quotations of the participant's perspecitves[55].

The sample size in this pilot study is limited to 30 participants and therefore lacks statistical power for quantitative analysis of the secondary outcomes.

ETHICS AND DISSEMINATION

Ethics

The study approval has been granted by the North East–Newcastle and North Tyneside Research Ethic Committee 1 (REC reference number: 18/NE/0265), and Research and Development (R&D) of the Northumbria Healthcare NHS Foundation Trust (Sponsor).

For peer review only

The study will be conducted in accordance with the principles of the International Conference for Harmonisation of Good Clinical Practice (European Medicines Agency, 2002). We have amended inclusion criteria for the study, and allowed the inclusion of individuals who have GS or walking speed above the EWGSOP cut-offs[31].

The study is funded by the National Institute for Health Research Newcastle Biomedical Research Centre, Newcastle University. Arla® will provide milk and scientific support related to this nutritional intervention. This study is registered online at https://www.isrctn.com/ISRCTN13398279.

Data monitoring

Throughout the study, the principal investigator (AG) will monitor recruitment, retention and compliance figures with the core research team (AG, CH, LD, TA). The core team will meet regularly to plan and evaluate study's day-to-day activities. Monthly meetings will be organised with the co-investigators (KD, ES, AAS) to update on study management and progress. The core research team and co-investigators will prepare consents, assessments, study protocol, and standard operating procedures for: (1) assessments and data reporting; (2) data management; (3) adverse events management and reporting, and (4) staff health risk assessment and safety procedures.

Adverse events

This is a low risk study. There is a small chance of transient muscle soreness, gastrointestinal discomfort, metabolic changes, and change in appetite. The chief investigator (TA) is clinically trained to oversee the research process, and the research team is trained in health and safety procedures during data collection. Each participant will be closely monitored and asked about any adverse events occurring at home or in the gym. Any suspected adverse events will be reported to the chief investigator (TA), who will also offer clinical oversight of the study. Any serious adverse events, as evaluated by TA thought to be related to the intervention, will be reported immediately to the study sponsor and

For peer review only

relevant ethics committee. Because of the low risk of adverse events, no independent Data Monitoring and Safety Committee will be appointed for this pilot study. The NHS indemnity insurance scheme will apply to cover the potential legal liability cover for harm to participants arising from the research. North Tyneside Council has the public and product liability cover for any potential harm arising from the fitness facility and equipment.

Data management

Data will be collected and managed in accordance with the EU General Data Protection Regulation (2018). At consent, participants will be assigned a unique study ID that will be used to pseudonymise primary research data collected from interviews and intervention. Identifiable data will be stored separately and will be accessible only to members of the research team who have additional research passport checks approved as part of their research role. Pseudonymised paper-based assessments will be double data entered, and all study data will be stored on secure, fire-wall and password protected servers of Newcastle University for 5 years.

Data Statement

Technical appendix, statistical code, and dataset will be available from the AGE Research Group data manager.

Dissemination, and Patient and Public Involvement

The following key outputs will contribute to study dissemination and impact. The results of the study will be reported to the funder (NIHR Newcastle Biomedical Research Centre). The funder, sponsor and industry support (Arla®) will have no role in the study design, conduct, data analysis, results interpretation, or writing. The aim is that at least two peer-reviewed papers will be published in high impact open access journals, and the results will be presented at relevant scientific conferences. A lay summary of the main results will be

For peer review only

presented to interested participants at a Public and Patient Involvement event. A flyer featuring the main results of the study and, if desired, an individual report titled 'My muscle function and strength before and after MIlkMAN' will be prepared for all study participants. Reports with abnormal results (blood pressure, BMI, fat mass, MMSE, and GDS) will be send to general practices. Regular updates on the study progress will be reported on a publicly accessible website.

Acknowledgements The authors would like to thank the North East and North Cumbria Clinical Research Network.

Funding This project is funded by the National Institute for Health Research (NIHR) Newcastle Biomedical Research Centre (reference number: BH Ref 173606 / PDB053), Newcastle University and supported by Arla® (in-kind (milk) and scientific advice about nutrition).

Author contribution AG, KD, ES, AAS, TR, LD, and CH developed and refined the study protocol. AG, KD, ES, AAS, and TA were responsible for study conception and design. AG drafted the manuscript. All co-authors revised the manuscript draft. AG was responsible for the analysis plan. All authors were responsible for critical revision and approved the final version of the manuscript.

Funding This project is funded by the National Institute for Health Research (NIHR) Newcastle Biomedical Research Centre (reference number: BH Ref 173606 / PDB053), Newcastle University and supported by Arla® (in-kind milk contribution).

Disclaimer The views expressed are those of the authors and not necessarily those of the NHS or NIHR.

Competing interest This study received 'in-kind' contribution from Arla®.

Ethics approval The North East – Newcastle and North Tyneside Research Ethic Committee 1 (REC reference number: 18/NE/0265).

Provenance and peer review Not commissioned; internally peer reviewed

Open Access This is an open access article distributed in accordance with the Creative Commons Attribution Non Commercial (CC BY-NC 4.0) license, which permits others to distribute, remix, adapt, build upon this work non-commercially, and license their derivative works on different terms, provided

For peer review only

that the original work is properly cited, appropriate credit is given, and changes made indicated, and the use is non-commercial. See: http://creaticecommons.org/license/by/4.0/

For peer review only

REFERENCES

1. Office for National Statistics. *Population Estimates for UK, England and Wales, Scotland and Northern Ireland: mid-2016.* https://www.ons.gov.uk/peoplepopulationandcommunity/populationandmigration/populationestimates/bulletins/annualmidyearpopulationestimates/mid2017 (accessed on 10 January 2019).
2. Rowe JW, Kahn RL. Successful aging 2.0: conceptual expansions for the 21st century. *J Gerontol B Psychol Sci Soc Sci* 2015;70:593–6.
3. Drewnowski A, Evans WJ. Nutrition, physical activity, and quality of life in older adults: summary. *J Gerontol A Biol Sci Med Sci* 2001;56:89–94.
4. Cruz-Jentoft AJ, Bahat G, Bauer J, et al. Sarcopenia: revised European consensus on definition and diagnosis. *Age Ageing* 2018 Oct 12. doi: 10.1093/ageing/afy169. [Epub ahead of print]
5. Janssen I, Ross R. Linking age-related changes in skeletal muscle mass and composition with metabolism and disease. *J Nutr Health Aging* 2005;9:408–19.
6. Visser, M.; Schaap, L.A. Consequences of sarcopenia. *Clin Geriatr Med* 2011;27: 387–399.
7. Manrique-Espinoza B, Salinas-Rodríguez, A, et al. Sarcopenia is associated with physical and mental components of health-related quality of life in older adults. *J Am Med Dir Assoc* 2017;18:636.e1–636.e5.
8. Mithal A, Bonjour JP, Boonen, S, et al. Impact of nutrition on muscle mass, strength, and performance in older adults. *Osteoporos Int* 2013;24:1555–66.
9. Marzetti E, Calvani R, Tosato M, et al. Physical activity and exercise as countermeasures to physical frailty and sarcopenia. *Aging Clin Exp Res* 2017;29: 35–42.
10. Cruz-Jentoft AJ, Sayer AA. Sarcopenia. *Lancet* 2019;393:2636–46.
11. Cruz-Jentoft AJ, Landi F, Schneider SM, et al. Prevalence of and interventions for sarcopenia in ageing adults: a systematic review. Report of the International Sarcopenia Initiative (EWGSOP and IWGS). *Age Ageing* 2014;43:748–59.
12. Pinedo-Villanueva R, Westbury LD, Syddall HE, et al. Health care costs associated with muscle weakness: A UK population-based estimate. *Calcif Tissue Int* 2018 Sep 22. doi: 10.1007/s00223-018-0478-1. [Epub ahead of print]
13. Volaklis KA, Halle M, Thorand B, et al. Handgrip strength is inversely and independently associated with multimorbidity among older women: Results from the KORA-Age study. *Eur J Intern Med* 2016, 31, 35–40.
14. Tsekoura M, Kastrinis A, Katsoulaki M, Billis E, Gliatis J. Sarcopenia and its impact on quality of life. *Adv Exp Med Biol* 2017;987:213–8.
15. Churchward-Venne TA, Holwerda AM, Phillips SM, et al. What is the optimal amount of protein to support post-exercise skeletal muscle reconditioning in the older adult? *Sports Med* 2016;46:1205–12.
16. Peterson M, Sen A, Gordon P. Influence of resistance exercise on lean body mass in aging adults: a meta-analysis. *Med Sci Sports Exerc* 2011;43:249–58.
17. Pennings B, Koopman R, Beelen M, et al. Exercising before protein intake allows for greater use of dietary protein–derived amino acids for de novo muscle protein synthesis in both young and elderly men. *Am J Clin Nutr* 2011;93:322–31.
18. Witard OC, Jackman SR, Breen L, et al. Myofibrillar muscle protein synthesis rates subsequent to a meal in response to increasing doses of whey protein at rest and after resistance exercise. *Am J Clin Nutr* 2014;99:86–95.

For peer review only - http://bmjopen.bmj.com/site/about/guidelines.xhtml

19. Moore DR, Churchward-Venne TA, Witard O, et al. Protein ingestion to stimulate myofibrillar protein synthesis requires greater relative protein intakes in healthy older versus younger men. *J Gerontol A Biol Sci Med Sci* 2015;70:57–62.
20. Tieland M, Dirks ML, van der Zwaluw N, et al. Protein supplementation increases muscle mass gain during prolonged resistance-type exercise training in frail elderly people: a randomized, double-blind, placebo-controlled trial. *J Am Med Dir Assoc* 2012;13:713–9.
21. Millward DJ. Nutrition and sarcopenia: evidence for an interaction. *Proc Nutr Soc* 2012;71:566–75.
22. Appleton KM. Barriers to and facilitators of the consumption of animal-based protein-rich foods in older adults. *Nutrients* 2016;8:187.
23. Pereira PC. Milk nutritional composition and its role in human health. *Nutrition* 2014;30:619–27.
24. Naclerio F, Larumbe-Zabala E. Effects of whey protein alone or as part of a multi-ingredient formulation on strength, fat-free mass, or lean body mass in resistance-trained individuals: a meta-analysis. *Sports Med* 2016;46:125–37.
25. Tang JE, Moore DR, Kujbida GW, Tarnopolsky MA, Phillips SM. Ingestion of whey hydrolysate, casein, or soy protein isolate: effects on mixed muscle protein synthesis at rest and following resistance exercise in young men. *J Appl Physiol (1985)* 2009;107:987–92.
26. Yang Y, Breen L, Burd NA, et al. Resistance exercise enhances myofibrillar protein synthesis with graded intakes of whey protein in older men. *Br J Nutr* 2012;108:1780–8.
27. Elliot TA, Cree MG, Sanford AP, et al. Milk ingestion stimulates net muscle protein synthesis following resistance exercise. *Med Sci Sports Exerc* 2006;38:667–74.
28. Rankin P, Stevenson EJ, Cockburn E. The effect of milk on the attenuation of exercise-induced muscle damage in males and females. *Eur J Appl Physiol* 2015;115:1245–61.
29. Rankin P, Landy A, Stevenson E, et al. Milk: an effective recovery drink for female athletes. *Nutrients* 2018;10(2).
30. Malmstrom TK, Morley JE. SARC-F: a simple questionnaire to rapidly diagnose sarcopenia. *J Am Med Dir Assoc* 2013;14:531–2.
31. Cruz-Jentoft AJ, Baeyens JP, Bauer JM, et al.; European Working Group on Sarcopenia in Older People. Sarcopenia: European consensus on definition and diagnosis: Report of the European Working Group on Sarcopenia in Older People. *Age Ageing* 2010;39:412–23.
32. Guralnik JM, Simonsick EM, Ferrucci L, et al. A short physical performance battery assessing lower extremity function: association with self-reported disability and prediction of mortality and nursing home admission. *J Gerontol* 1994;49:M85–94.
33. Moon JR, Stout JR, Smith-Ryan AE, et al. Tracking fat-free mass changes in elderly men and women using single-frequency bioimpedance and dual-energy X-ray absorptiometry: a four-compartment model comparison. *Eur J Clin Nutr* 2013;67 Suppl 1:S40–6.
34. Roberts HC, Denison HJ, Martin HJ, et al. A review of the measurement of grip strength in clinical and epidemiological studies: towards a standardised approach. *Age Ageing* 2011;40:423–9.

For peer review only

35. Gandek B, Ware JE, Aaronson NK, et al. Cross-validation of item selection and scoring for the SF-12 Health Survey in nine countries: results from the IQOLA Project. International Quality of Life Assessment. *J Clin Epidemiol* 1998;51:1171–8.
36. Wade DT, Collin C. The Barthel ADL Index: a standard measure of physical disability? *Int Disabil Stud* 1988;10:64–7.
37. Saghaei, M, Saghaei, S. Implementation of an open-source customizable minimization program for allocation of patients to parallel groups in clinical trials. *J Biomed Sci Eng* 2011;4:734–9.
38. Moore CG, Carter RE, Nietert PJ, Stewart PW. Recommendations for planning pilot studies in clinical and translational research. *Clin Transl Sci* 2011;4(5):332-7.
39. Chandola T, Jenkinson C. The new UK National Statistics Socio-Economic Classification (NS-SEC); investigating social class differences in self-reported health status. *J Public Health Med* 2000;22:182–90.
40. Smith T, Noble M, Noble S, et al. *The English Indices of Deprivation 2015*. London: Department of Communities and Local Government; 2015. https://assets.publishing.service.gov.uk/government/uploads/system/uploads/attachment_data/file/464485/English_Indices_of_Deprivation_2015_-_Technical-Report.pdf. Accessed April 12, 2018.
41. Brown LM, Schinka JA. Development and initial validation of a 15-item informant version of the Geriatric Depression Scale. *Int J Geriatr Psychiatry* 2005;20:911–8.
42. Simpson E, Bradley J, Poliakov I, et al. Iterative development of an online dietary recall tool: INTAKE24. *Nutrients* 2017;9(2).
43. Wilson MM, Thomas DR, Rubenstein LZ, et al. Appetite assessment: simple appetite questionnaire predicts weight loss in community-dwelling adults and nursing home residents. *Am J Clin Nutr* 2005;82:1074–81.
44. Collerton J, Barrass K, Bond J, et al. The Newcastle 85+ study: biological, clinical and psychological factors associated with healthy ageing: study protocol. *BMC Geriatr* 2007;7:14.
45. Robinson MM, Dasari S, Konopka AR, et al. Enhanced protein translation underlies improved metabolic and physical adaptations to different exercise training modes in young and old humans. *Cell Metab* 2017;25:581–92.
46. Peterson MD, Gordon PM. Resistance exercise for the aging adult: clinical implications and prescription guidelines. *Am J Med* 2011;124:194–8.
47. Borde R, Hortobágyi T, Granacher U. Dose-response relationships of resistance training in healthy old adults: a systematic review and meta-analysis. *Sports Med* 2015;45:1693–720.
48. Brzycki, M. Strength testing—predicting a one-rep max from reps-to-fatigue. *J Phys Educ Recreat Dance* 1993;64:88–90.

49. Borg E, Borg G. A comparison of AME and CR100 for scaling perceived exertion. *Acta Psychol (Amst)* 2002;109:157–5.
50. Hurst C, Weston KL, Weston M. The effect of 12 weeks of combined upper-and lower-body high-intensity interval training on muscular and cardiorespiratory fitness in older adults. *Aging Clin Exp Res* 2019;31:661–71.

51. Whelton PK, Carey RM, Aronow WS, et al. 2017 ACC/AHA/AAPA/ABC/ACPM/AGS/APhA/ASH/ASPC/NMA/PCNA Guideline for the Prevention, Detection, Evaluation, and Management of High Blood Pressure in Adults: Executive Summary: A Report of the American College of

For peer review only - http://bmjopen.bmj.com/site/about/guidelines.xhtml

Cardiology/American Heart Association Task Force on Clinical Practice Guidelines. *Circulation* 2018;138:e426–83.
52. Rezk CC, Marrache RC, Tinucci T, et al. Post-resistance exercise hypotension, hemodynamics, and heart rate variability: influence of exercise intensity. *Eur J Appl Physiol* 2006;98:105–12.
53. Huggett DL, Elliott ID, Overend TJ, et al. Comparison of heart-rate and blood-pressure increases during isokinetic eccentric versus isometric exercise in older adults. *J Aging Phys Act* 2004;12:157–69.
54. The Food Standards Agency (2002). *McCance and Widdowson's the Composition of Foods Integral Dataset* (6th Summary Edition). http://webarchive.nationalarchives.gov.uk. Accessed in: August 20, 2017
55. Hsieh HF, Shannon SE. Three approaches to qualitative content analysis. *Qual Health Res* 2005;15:1277–88.

For peer review only

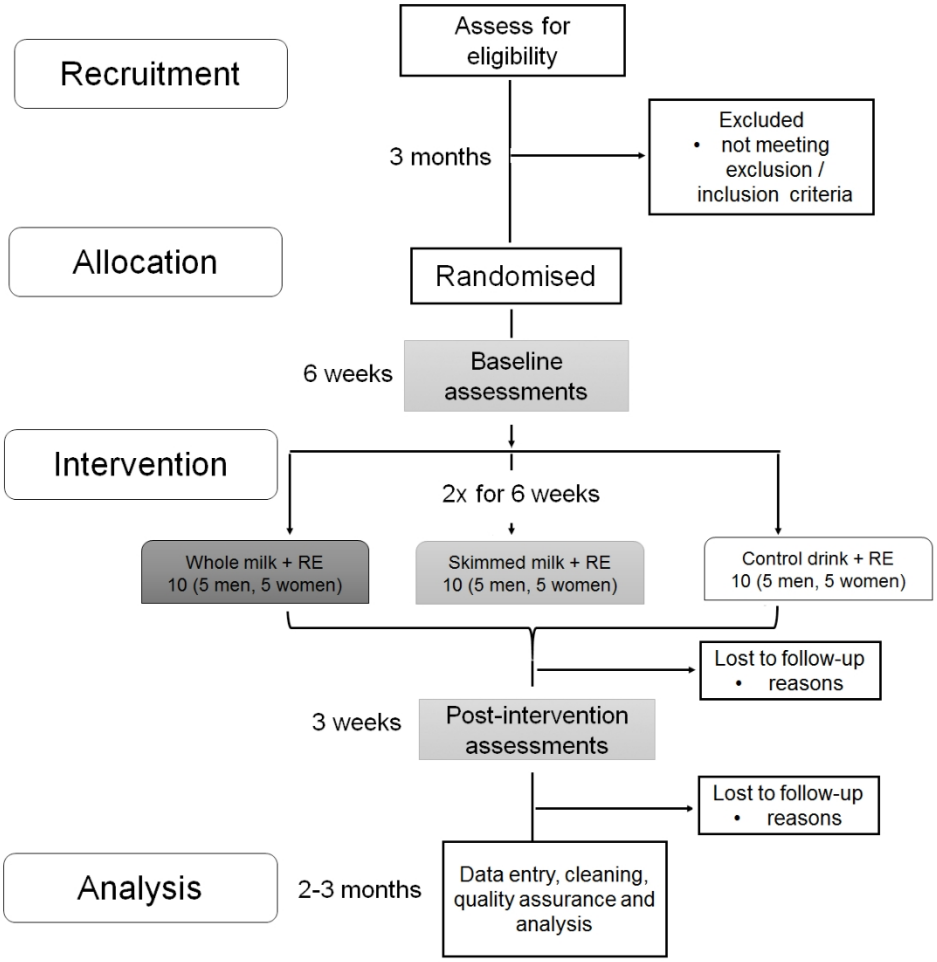

Figure 1. Study flow chart.

109x111mm (300 x 300 DPI)

SUPPLEMENTARY MATERIAL

Appendix 1

THE SARC-F QUESTIONNAIRE[30]

Strength: How much difficulty do you have in lifting and carrying 10 pounds / a bag of shopping?
None: 0
Some: 1
A lot or unable: 2

Assistance in walking: How much difficulty do you have walking across a room?
None: 0
Some: 1
A lot, use aids, or unable: 2

Rise from a chair: How much difficulty do you have transferring from a chair or bed?
None: 0
Some: 1
A lot or unable without help: 2

Climb stairs: How much difficulty do you have climbing a flight of 10 stairs?
None: 0
Some: 1
A lot or unable: 2

Falls: How many times have you fallen in the past year?
None: 0
1 to 3 falls: 1
4 or more falls: 2

For peer review only

Appendix 2

CR100 scale[49]

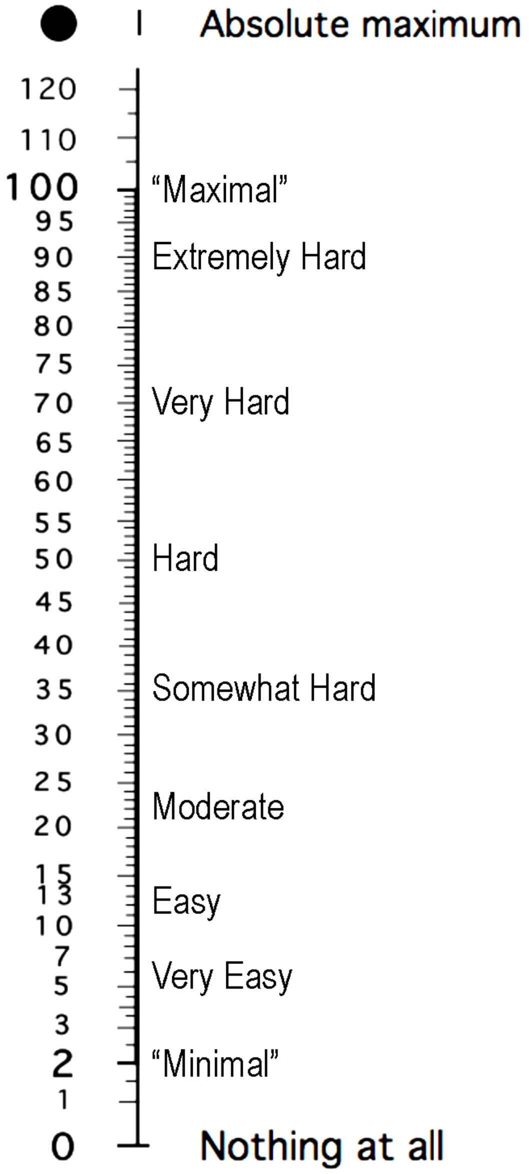

Instructions for participants (explained to participants by exercise physiologist)
Assessment ~10 minutes after exercise

- Using the scale, we would like you to rate your perceptions of EFFORT (RPE), that is, how difficult the session felt to you.
- Your perception of EFFORT should be a conscious awareness of how hard (or easy) the whole session was. It should not be influenced your feelings of fatigue, pain or discomfort (try separating these from effort as best you can).
- You will be asked to rate your perceptions of overall effort (the whole session after the warm-up), and then separate scores for upper-body muscle effort (arm muscles) and lower-body muscle effort (leg muscles).

Appendix 3

Visual analogue scale for muscle soreness

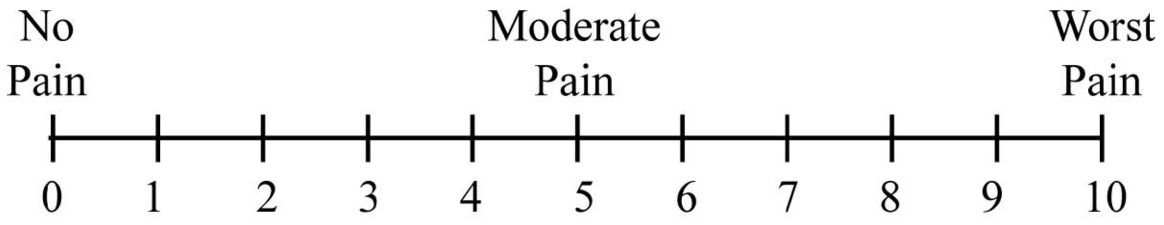

0 = no pain, 1-3 = mild pain, 4-6 = moderate pain; 7-10 = severe

Instructions for participants (explained to participants by a researcher)

- This is a Visual Analogue Scale. The scale describes the intensity of your muscle soreness, 0 meaning no pain at all, 1 to 3 meaning mild pain, 4-6 moderate pain, and 7-10 the worst pain.
- Assessment ~45 minutes after exercise: Which number on the scale describes the best your muscle soreness in your arms and legs?
- *Assessment in the evening over the telephone (6-7 hours after exercise)*: A researcher will call you in the evening after each visit to the gym to ask you again about your muscle soreness in your arms and legs. Please use this visual analogue scale to rate your muscle soreness in your arms and legs.

For peer review only

BMJ Open

Milk and resistance exercise intervention to improve muscle function in community-dwelling older adults at risk of sarcopenia (MIlkMAN): protocol for a pilot study

Journal:	*BMJ Open*
Manuscript ID	bmjopen-2019-031048.R2
Article Type:	Protocol
Date Submitted by the Author:	02-Sep-2019
Complete List of Authors:	Granic, Antoneta; Newcastle University, Institute of Neuroscience<br>Hurst , Christopher; Newcastle University, Institute of Neuroscience<br>Dismore , Lorelle ; Newcastle University, Institute of Neuroscience<br>Davies , Karen ; Newcastle University, Institute of Neuroscience<br>Stevenson , Emma ; Newcastle University, Institute of Cellular Medicine<br>Sayer, Avan; Newcastle Upon Tyne Hospitals NHS Foundation Trust, NIHR Biomedical Research Centre; Newcastle University<br>Aspray , Terry ; Newcastle University, Institute of Cellular Medicine
**Primary Subject Heading**:	Geriatric medicine
Secondary Subject Heading:	Nutrition and metabolism, Patient-centred medicine
Keywords:	pilot study, older adults, sarcopenia, physical function, whole milk, resistance exercise

SCHOLARONE™
Manuscripts

Milk and resistance exercise intervention to improve muscle function in community-dwelling older adults at risk of sarcopenia (MIlkMAN): protocol for a pilot study

Antoneta Granic[1-3*], Christopher Hurst[1,2†], Lorelle Dismore[1,2†], Karen Davies[1-3], Emma Stevenson[4,5], Avan A Sayer[1-3], Terry Aspray[1,2,4, 6]

[1]AGE Research Group, Institute of Neuroscience, Newcastle University, Newcastle upon Tyne, United Kingdom

[2]NIHR Newcastle Biomedical Research Centre, Newcastle upon Tyne Hospitals NHS Foundation Trust and Newcastle University, Newcastle upon Tyne, United Kingdom

[3]Newcastle University Institute for Ageing, Newcastle upon Tyne, United Kingdom

[4]Institute of Cellular Medicine, Newcastle University, Newcastle upon Tyne, United Kingdom

[5]Human Nutrition Research Centre, Newcastle University, Newcastle upon Tyne, United Kingdom

[6]Newcastle upon Tyne Hospitals NHS Foundation Trust, Musculoskeletal Unit, Freeman Hospital, Newcastle upon Tyne, United Kingdom

[†]equal contribution

For peer review only

*correspondence:

E-mail: antoneta.granic@newcastle.ac.uk

Phone: +44 (0) 1912081112

Biomedical Research Building, 1st Floor

Campus for Ageing and Vitality

Newcastle University

Newcastle upon Tyne, NE4 5PL

United Kingdom

Word count: 5,725

For peer review only

ABSTRACT

Introduction: Sarcopenia is a progressive muscle disorder characterised by decline in skeletal muscle mass, strength and function leading to adverse health outcomes, including falls, frailty, poor quality of life, and death. It occurs more commonly in older people and can be accelerated by poor diet and low physical activity. Intervention studies incorporating higher dietary protein intakes or protein supplementation combined with resistance exercise (RE) have been shown to limit muscle function decline. However, less is known about the role of whole foods in reducing the risk of sarcopenia. Milk is a source of high-quality nutrients, which may be beneficial for skeletal muscle. This pilot study examines the feasibility and acceptability of milk consumption with RE to improve muscle function in community-dwelling older adults at risk of sarcopenia.

Methods and Analysis: 30 older adults aged ≥65 years will be randomly allocated to three groups: 'whole milk + RE', 'skimmed milk + RE' or 'control drink + RE'. Assessments will take place in participants' homes, including screening (milk allergies, grip strength, walking speed), baseline and post-intervention health and function. All participants will undertake a structured RE intervention twice/week for 6 weeks at a local gym, followed by the consumption of 500ml of whole or skimmed milk (each ~20 g of protein) or an isocaloric control drink and another 500ml at home. Participants' views about the study will be assessed using standardised open-ended questions. The primary outcomes include feasibility and acceptability of the intervention with recruitment, retention, and intervention response rates. Analyses will include descriptive statistics, exploration of qualitative themes and intervention fidelity.

Ethics and dissemination: The North East–Newcastle and North Tyneside Research Ethic Committee 1 (18/NE/0265) approved the study. Outputs include pilot data to support funding applications; public involvement events; presentation at conferences, and peer-reviewed publication.

Trail registration number: ISRCTN13398279; Pre-results.

For peer review only

Keywords: pilot study, older adults, physical function, muscle strength, sarcopenia, whole milk, resistance exercise, dietary protein, quality of life

Strengths and limitations of this study

- To our knowledge, this is the first pilot study examining the feasibility and acceptability of the whole versus skimmed milk with resistance exercise (RE) intervention in community-dwelling older adults living in the UK.
- The intervention is conducted in a local gym that is easily accessible to older adults who will benefit from the familiarisation with RE programme conducted in the community to foster continuous engagement.
- Post-intervention interview will allow for the collection of qualitative evidence to support planned future trial, including better understanding of the barriers and facilitators of community-based intervention.
- Because this an evaluation of a pilot implementation, the sample size is not based on statistical power.
- Although we do not anticipate any definite results in exploring differences between intervention groups, the results will be used to aid power calculations for planned future substantive research.

only

INTRODUCTION

The UK population is ageing rapidly; the number of adults aged ⩾65 increased by 17.3% in the last decade, and in mid-2017 was estimated to account for 18.2% of the total population of 66 million[1]. Understanding factors associated with healthy ageing[2] such as diet and physical activity[3] for optimising health and wellbeing of an ageing population is essential for the development of effective interventions.

Sarcopenia is a progressive, generalised muscle disorder characterised by decline in skeletal muscle mass, strength and function[4], which leads to an increased risk of falls, frailty, disability, low health-related quality of life (QoL) and death[5-8]. It occurs more commonly in older people and can be accelerated by poor diet and low physical activity[8,9]. The prevalence of sarcopenia increases with advancing age—and although dependent on the algorithm used to define sarcopenia[10]—it reaches more than 20% in men and women aged ≥85 years[11], resulting in an estimated excess of health care cost of £2.5 billion/year in the UK[12]. This emphasises the need for sustainable preventive measures aimed to preserve and optimise muscle health and function in a rapidly ageing population before the onset of difficulties leading to or exacerbating the risk of sarcopenia.

Protein intake and exercise for healthy muscle ageing

Loss of muscle mass and strength can be accelerated by poor diet, low levels of physical activity and the presence of long-term conditions[8,9,13], leading to diminished QoL[7,14]. Adequate intake of dietary protein and resistance exercise (RE) are recognised as effective interventions to promote skeletal muscle health and reduction of physical decline[15,16]. Specifically, intervention studies that examined a combined effect of protein supplements, comprising essential amino acids (EAA) and RE to stimulate muscle protein synthesis (MPS) have observed an increase in total muscle protein within 3-5 hours following exercise in both young and older adults[17]. Compared with young adults, older adults experience a blunted response after protein ingestion to stimulate MPS (anabolic resistance), especially in

For peer review only

response to lower amounts of protein or EAA of <20g or <10g, respectively. Other studies have shown that greater amounts of protein supplementation and intermittent feeding in combination with repeated bouts of RE resulted in increased muscle mass in older adults, even in those diagnosed with frailty and sarcopenia[18-20].

However, there is limited research on the role of whole foods rich in protein (e.g. milk and dairy products, fish, and meats) in maintaining skeletal muscle mass, strength and function in older adults at risk of sarcopenia. Regular consumption of high-quality, nutrient-dense foods, high in macro- and micronutrients relevant for muscle[21] within a varied diet may provide a platform for developing strategies for maintenance of muscle health and function in later life that do not include supplements and medical products, and may be easier adopted as a behavioural change in older adults[22].

Milk for muscle health: current evidence and why this pilot is needed

Cow's milk is an example of a whole food with the potential to ameliorate loss of skeletal muscle mass, strength and function. Whole milk (3.6% fat) is a source of high-quality proteins (whey and caseins), minerals (e.g. calcium, phosphorus, magnesium), vitamins (e.g. A, B, D, and E), carbohydrates, bioactive lipids and fatty acids (mono- and polyunsaturated, and saturated fatty acids)[23]. Whey protein is considered superior to other protein sources for MPS after exercise in younger and older adults because of its greater bioavailability and solubility, and higher content of the branched-chain amino acids, including leucine[24-26]. Furthermore, the concurrent intake of milk fats with protein in whole milk has been shown to increase the use of EAA for MPS after exercise in young men compared with skimmed milk (0.3% fat)[27], suggesting additional benefits of milk lipids for muscle. Other benefits of milk containing fat include reduction in exercise-related muscle damage, soreness, and decline in muscle performance in young adults and athletes[28,29] compared with energy–matched (isocaloric) carbohydrate drink. However, little is known about the effect of milk and protein-fat ratio in milk on muscle in older adults, particularly the impact on

For peer review only

muscle function of varying milk fat contents (whole versus skimmed) providing >20g protein/day after exercise.

We hypothesised that whole milk (3.6% fat), providing >20g of protein and the same amount of energy as fat and protein-free carbohydrate drink, after structured exercise conducted in the community may be a feasible and acceptable intervention for maintaining skeletal muscle mass, strength and function in older adults at risk of sarcopenia.

Study aims

The primary aims are:

(1) To examine the feasibility and acceptability of whole (3.6% fat) or skimmed milk (0.3% fat) in combination with RE as an intervention in community-dwelling older adults aged ≥65 at risk of sarcopenia. This aim will answer the following questions: Is an intervention of 2 × 500ml milk + RE twice/week for 6 weeks (a) feasible and (b) acceptable to older adults?

(2) To provide essential data for planned future substantive research.

The secondary aim of the study will be to explore whether consumption of whole or skimmed milk + RE has an influence on physical performance, muscle mass, strength and self-reported QoL in older adults at risk of sarcopenia.

For peer review only

METHODS AND ANALYSIS

Study design

This is a pilot study with a parallel group design involving 30 participants (aiming for 15 men and 15 women) aged ≥65 who will be randomised into three intervention groups: (group 1) 'whole milk + RE'; (group 2) 'skimmed milk + RE', and (group 3) 'control drink' + RE. Data will be collected from: (1) health and functioning assessments (screening, baseline and post-intervention interview); (2) the nutrition + exercise intervention over 6 weeks, and (3) participants' feedback about the study.

Exclusion and inclusion criteria

The study will include older adults who are registered patients with General Practitioner (GP) practices within the National Institute for Health Research (NIHR) North East and North Cumbria Clinical Research Network (CRN), United Kingdom. Table 1 lists the inclusion and exclusion criteria that are applied to patient database searches, performed in GP practices and screening interviews conducted by the research team.

Table 1. Inclusion and exclusion criteria for the MIlkMAN: pilot

Criteria	Patient database searches	Screening interview
Inclusion	aged 65 and over	
	live in the community	
Exclusion	diabetes mellitus type 1 or type 2	lacks capacity to consent to participate
	chronic kidney disease stage 4 or 5 (estimated glomerular filtration rate <30ml / min /1.73m^2)	lactose intolerance
	liver function impairment (AST >2.5 times upper limit of normal range within the last 6 months)	dislikes milk or cranberry juice (control drink)
	chronic lung disease requiring maintenance steroid therapy (e.g. COPD, severe asthma)	participated in a structured RE training and gym programme in the last month
	end-stage terminal illness	dislikes gym exercise with equipment
	cardiac pacemaker or severe heart failure or other significant heart disease	unintentional weight loss ⩾5kg in the last 3 months
	uncontrolled hypertension (>160/100 mmHg) and uncontrolled hypotension (<100 mmHg systolic) within last 6 months	unable to understand instructions for muscle strength and function assessments in English or unwilling to participate in protocol when explained
	hip or knee replacement	an individual who the research team (exercise physiologist) evaluates as not suitable for the intervention because of safety reasons
	impaired mobility (unable to walk without an aid including wheelchair	
	current prescription of warfarin (potential interference with control drink)	
	BMI ⩾30kg/m2	
	an individual who the GP feels it is inappropriate for the research team to	

For peer review only

	approach for safety reasons: any medical and physical conditions that preclude safe participation in a RE programme (long-term conditions likely to lead impaired function over 6 months)

AST, Aspartate Aminotransferase; BMI, body mass index; COPD, Chronic Obstructive Pulmonary Disease; GP, general practitioner.

Study population and recruitment

Primary care recruitment will be carried out with the assistance of North East and North Cumbria CRN, England, which provides support with access to general practices and their patients. Two practices in North Tyneside Clinical Commissioning Group were identified for feasibility using exclusion/inclusion criteria (Table 1, left column) and provided feedback during the application for funding in 2018.

Recruitment will be organised in two stages: pre-screening (GP practices) and screening (research team). At the pre-screening stage, practices will identify potential participants from their patient database using exclusion/ inclusion criteria (Table 1, left column), and then mail out recruitment packs, containing detailed information about the study with a reply slip. Interested individuals will be interviewed over the telephone by a researcher using a 5-item SARC-F questionnaire (Appendix 1)[30] to assess any difficulties with day-to-day activities (lifting and carrying 10 pounds, walking, rising from a chair and climbing stairs), and number of falls in the past year. Those evaluated by the research team to have no major difficulties that would preclude safe participation in the exercise programme (e.g. unable to walk across a room), will be visited in their own home for a screening interview to obtain written informed consent, and to evaluate inclusion/exclusion criteria not screened through GPs (Table 1, right column).

Those who meet the criteria will be assessed further for muscle strength (grip strength, GS) and function (walking speed) based on the following cut-offs: <20 kg (women), and <30 kg (men)[31] for low GS; and <0.8 m/s or ⩾5 s over 4 m distance[31] for low walking speed. GS measurements (high or low) at the screening interview will be used for

For peer review only - http://bmjopen.bmj.com/site/about/guidelines.xhtml

minimisation along with sex to allow equal distribution of those with muscle strength weakness across the intervention groups. However, the target number of those with 'low' GS will not be established a priori. Therefore, the study will recruit older adults with some deficits in muscle health and those without for whom it is determined to be safe to participate in the study (primary aims), and hypothesised to benefit from the intervention regardless of the deficits (secondary aims).

Study outcomes

Table 2 lists the primary and secondary study outcomes and when they are completed.

Table 2. Study outcome measures

Measure	Screening	Baseline	Post-intervention
Primary			
Feasibility and acceptability of intervention in a local gym setting			×
Applicability			×
Dosage and duration of intervention			×
Compliance		×	×
Attrition		×	×
Adverse health effects			×
Response rates to questionnaires, assessments, and intervention	×	×	×
Secondary			
Short Physical Performance Battery[32] (balance, 4m-gait speed, 5-chair stands)		×	×
Muscle mass[33]		×	×
Grip strengh[34]	×	×	×
SF-12 Health Survey[35]		×	×
Barthel Index[36]		×	×

The secondary outcome measures will explore differences in physical performance measures between the groups, pre and post-intervention. GS (muscle strength)[34] will be expressed as the maximum reading of 6 trials of both hands using a Jamar hand-held 5030J1 dynamometer. Body composition (muscle mass) will be measured using Bioelectric Impedance Analysis[33] (BIA; Tanita MC-780MA Body Composition Analyzer). Self-reported QoL will be measured using SF-12[35], and activities of daily living with Barthel Index[36].

For peer review only

Randomisation

A researcher will allocate thirty participants to one of the three interventions to ensure balanced allocation of participants between the groups based on sex and muscle strength (GS at screening assessment) using a free, open-source minimisation software (MiniPy 0.3, http://minimpy.sourceforge.net)[37]. The software features elements of randomness in the minimisation algorithm by allocating the first participant randomly into one of the interventions, and assigning the subsequent participants on hypothetical stepwise allocation to every group and computation of the imbalance score corresponding to each allocation. The imbalance scores are compared and participants allocated to the group corresponding to the least imbalance score (preferred group).

The sample size for the pilot is not based on statistical power but guided by the consideration to fulfil the primary aims of the study (e.g. provide guidelines for the larger trial) and practical feasibility[38].

Consent

Written informed consent will be obtained by a researcher visiting the participants during the home-based screening assessment prior to randomisation. Capacity to consent will be assessed using an established consent pathway. Throughout the active research phase (i.e. from baseline to post-intervention assessment), the notion of process consent will be implemented, requiring an ongoing exchange of information about the study and confirming the participants willingness to proceed, ensuring that participants are free to reconsider and withdraw from the study at any time. If a participant loses capacity during the research process, he/she will be withdrawn from the study.

Flow diagram of the study

A flow diagram of the study protocol with timelines is outlined in Figure 1. The recruitment (assessment of eligibility) up to allocation (randomisation into 3 groups) will be

For peer review only

finalised within 3 months, followed by baseline assessments for health and functioning in participants' homes, and a 6-week intervention in a local gym.

>>Insert Figure 1<<

Figure 1. Study flow chart.

Home-based post-intervention assessments, including participants' feedback about the study, will be conducted the week following completion of the intervention, and finalised within 3 weeks. Data analysis will be completed after active data collection (from randomisation to post-intervention assessment). Data collection for each participant will span approximately 10 weeks: (1) week 1: consent and home-based screening assessment; (2) week 2: home-based baseline interview; (3) week 3 to week 8: 6-week intervention twice/week (12 visits at a local gym/sport centre); (4) week 10: post-intervention home-based interview. Except for the intervention (6 consecutive weeks), this time scale can be adjusted to participants' individual needs with a maximum 3 weeks gap between baseline assessment and the first week of intervention, and a maximum 3 weeks gap between the last week of intervention and post-intervention assessment.

Data collection

To determine the feasibility and acceptability of the study, the following data will be collected and analysed. The number of individuals approached; the reasons for not opting to take a part in the study (reported with permission); the recruitment and retention rates; the reasons for attrition; the completion of objective assessments and questionnaires; the number of RE sessions completed, and compliance with the milk/control drink intake. Other health and functioning data will be collected at the home-based screening and baseline interview, during the intervention (at the gym), and at the home-based post-intervention interview. Participants' attitudes and opinions about the study will be collected at the post-intervention interview using a combination of multiple-response and standardised open-ended questions.

For peer review only

Screening interview

Once potential participants have been identified by the GP practices, having expressed an interest in participation and being interviewed over the phone by a researcher, a mutually convenient appointment will be arranged for a screening visit at the participant's home. Participants will be screening for other exclusion/ inclusion criteria not assessed at by the GP practices (Table 1, right column), and to establish participants' muscle strength (GS) and functioning status (walking speed) based on the established cut-offs[31]. Informed consent will be obtained before any assessment is undertaken. Eligible individuals will be informed about the study procedure and their journey through the study (from randomisation to post-intervention assessment).

Baseline interview

Table 3 lists domains and assessments for the baseline interview with times needed to administer (in minutes). The detailed health and functioning profile will involve minimal risk and inconvenience to participants, and it will be conducted within 70 minutes (including breaks and excluding assessments done at the gym).

Table 3. Domains and assessments at baseline

Domain and assessment	Time to administer (min)
Sociodemographic profile	total: 6
age	
sex	
marital status	
education	
social class (NS-SEC)[39]	
deprivation (Multiple Index of Deprivation)[40]	
General health	total: 54
SF-12 Health Survey[35]	4
self-reported diseases diagnosed by a doctor	2
list of medication (prescribed and over-the-counter)	2
Mini Mental State Examination	10
Geriatric Depression Scale (15-item version)[41]	7
Barthel Index (Activities of Daily Living)[36]	3
blood pressure (systolic and diastolic)*	5
Intake24: 24-hr dietary recall[42]* (https://intake24.co.uk/)	20
appetite (a 4-item Simplified Nutritional Appetite Questionnaire)[43]*	1
Lifestyle	total: 5
self-reported physical activity[44]	3
smoking status	1

For peer review only

alcohol intake	1
Anthropometry	total: 14
demi-span	2
waist and hip circumference	3
calf circumference	2
muscle mass (body composition by BIA)[33]*	7
Physical functioning	total: 24
Short Physical Performance Battery (SPPB)[32]	10
balance (a side-by-side tandem; semi-tandem; tandem)	3
4-m gait speed	3
5-chair stands	4
maximum grip strength (measured 3 times in each arm)	4

*Assessments done at the intervention site (gym) before and after each RE session (blood pressure), and before the first RE session (diet, appetite, body composition).

Intervention

Resistance exercise (RE)

All participants will perform two RE sessions per week for 6 weeks at a community leisure centre (The Parks, North Tyneside Council, North Shields, UK) that is easily accessible and close to their residence. For each RE session a time slot of ~45-60 min in duration will be allocated, with a minimum of 48 hours between sessions. The sessions will be completed in groups of 2-4 participants under the supervision of an experienced exercise physiologist (CH). Exercise intensity, volume, frequency and duration have been determined based on recent literature[45-47] and the American College of Sports Medicine (ACSM) recommendations for older adults[46]. With the exception of the structured exercise sessions and the nutritional intervention prescribed, participants will be asked to maintain their usual diet, level of physical activity and lifestyle throughout the duration of the intervention period.

During the first RE session participants will be familiarised with the exercises (leg press, leg curl, seated row, chest press) as well as the equipment to be used throughout the intervention with correct technique demonstrated and extensively described. Following this, participants' one repetition maximum (1RM) will be estimated for all four exercises using a previously established equation[48].

Following the initial RE session, each remaining session will begin with a 5-minute warm-up performed at progressive intensity using either a cycle ergometer or treadmill.

For peer review only

Participants will then complete 2-4 sets of 8-12 repetitions at a workload of 70-79%1RM[45,46] for all four of the exercises listed above. Each session will conclude with a short cool-down period of low intensity aerobic exercise, and (except the initial session) will be completed within 30 minutes.

In an attempt to promote participants' engagement with RE, each will receive a training log with diagrams and short instructions with space to record the details of the exercise completed. Participants' gym attendance, sets and repetitions completed, and weight lifted will be recorded following each RE session allowing for the calculation of measures of training load (e.g. volume load [number of sets × number of repetitions × weight lifted]). In addition to measures of external training load, resistance training intensity will be monitored using participant ratings of perceived exertion. Using the CR100® scale[49] (Appendix 2), participants will provide an overall session rating of perceived exertion (sRPE) as well as differential ratings of perceived exertion for upper-body muscle exertion (RPE-U) and lower-body muscle exertion (RPE-L) approximately 10 minutes after the completion of each RE session[50]. Each participant must complete at least 10 sessions (out of 12) to be considered compliant with the exercise programme.

Blood pressure and heart rate will be measured pre and post each RE session in each participant and compared to the guidelines provided by the American College of Cardiology/American Heart Association Task Force[51] and existing literature[52,53]. Muscle soreness will be assessed using a simple visual analogue scale (Appendix 3) at ~40-45 minutes and at ~6-7 hours after each RE session.

Nutritional intervention

On average, 500ml milk contains ~20g of protein needed to stimulate MPS above stimulation provided by RE[17,18]. Whole cow milk (nutritional estimates of 22 UK samples during winter and summer) provides 66 kcal/100g of energy[54]. Arla Cravendale® whole milk contains 3.6g fat, 3.4g protein, and 4.7g of carbohydrates per 100g of milk. Arla

For peer review only

Cravendale® skimmed milk contains 0.3g fat, 3.6g protein, and 4.9g of carbohydrate per 100g of milk. The energy content of the control drink (cranberry juice; Ocean Spray Classic®; 23/kcal/100g of energy) will be balanced to match whole milk energy content and supplemented with maltodextrin (4kcal/g; www.myprotein.com) on the day of intervention. Both milk and juice will be provided in packs of 1L, bought fresh on a weekly basis through a local retailer and kept in a locked refrigerator at the Campus for Ageing and Vitality, Newcastle University.

The milk/control drink will be consumed as a bolus intake of 500ml under the supervision of a researcher immediately after exercise during the recovery period, aiming for complete consumption within ~45 minutes prior to leaving the centre. The second dose of 500ml will be consumed at the participants' home over the next 4-5 hours as a part of their usual diet with other foods. Participants' compliance with consumption of the milk/control drink will be checked in the evening (~6-7 hours post-exercise). Each participant will be provided with a plastic measuring jug (500ml) to measure their consumption at home and to report it back to a researcher over the telephone.

Post-intervention interview

Table 3 lists the domains and assessments that will be repeated at the post-intervention interview. Briefly, a home visit will be arranged with each participant after the 6-week intervention to assess his/her general health and physical functioning, including SPPB, muscle mass (body composition), muscle strength (GS), SF-12, Barthel Index, diet and appetite. Additionally, participants' feedback will be collected at the end of the post-intervention interview using a combination of structured multiple-response and standardised open-ended questions. The following themes will be explored: (1) attitudes and barriers to consuming of 2 × 500ml milk/control drink intake post-exercise (e.g. volume of liquid, taste, etc.); (2) opinion about milk as a functional food for muscle strength/ function; (3) changes in appetite and habitual diet because of milk/control drink intake, and (4) what was liked and disliked about the study (intervention), including motivations and barriers to continue

For peer review only

engagement in a local gym. The post-intervention interview will be completed within 50 minutes.

Statistical methods

As this is a feasibility and acceptability study aimed to inform a larger trial, the focus of data analysis will be descriptive. Using descriptive statistics (percentages, means (SDs)), we will calculate the response rates, the numbers consented and randomised, the retention rate, and the number, length and frequency of interviews and RE sessions. Compliance with the milk and control drink intervention will be calculated as a percentage of actual consumption divided by expected consumption over the 6-week intervention. Recording the number of repetitions for each exercise within each RE session and the weight lifted will allow calculation of several indices of training intensity. Mean and SDs (or equivalent) for questionnaire data and assessments will be reported at screening, baseline and post-intervention interview. Missing data will be recorded and evaluated.

Participants' experiences and views about the study will be assessed with standardised open-ended questions. This data will be analysed using content analysis[55]. Content analysis is a flexible method for analysing text data. Coding categories will be derived directly from the data and themes will be identified supported with relevant quotations of the participant's perspecitves[55].

The sample size in this pilot study is limited to 30 participants and therefore lacks statistical power for quantitative analysis of the secondary outcomes.

Dissemination, and Patient and Public Involvement

The following key outputs will contribute to study dissemination and impact. The results of the study will be reported to the funder (NIHR Newcastle Biomedical Research Centre). The funder, sponsor and industry support (Arla®) will have no role in the study design, conduct, data analysis, results interpretation, or writing. The aim is that at least two

For peer review only

peer-reviewed papers will be published in high impact open access journals, and the results will be presented at relevant scientific conferences. A lay summary of the main results will be presented to interested participants at a Public and Patient Involvement event. A flyer featuring the main results of the study and, if desired, an individual report titled ‘My muscle function and strength before and after MIlkMAN’ will be prepared for all study participants. Reports with abnormal results (blood pressure, BMI, fat mass, MMSE, and GDS) will be send to general practices. Regular updates on the study progress will be reported on a publicly accessible website.

ETHICS AND DISSEMINATION

Ethics

The study approval has been granted by the North East–Newcastle and North Tyneside Research Ethic Committee 1 (REC reference number: 18/NE/0265), and Research and Development (R&D) of the Northumbria Healthcare NHS Foundation Trust (Sponsor). The study will be conducted in accordance with the principles of the International Conference for Harmonisation of Good Clinical Practice (European Medicines Agency, 2002). We have amended inclusion criteria for the study, and allowed the inclusion of individuals who have GS or walking speed above the EWGSOP cut-offs[31].

The study is funded by the National Institute for Health Research Newcastle Biomedical Research Centre, Newcastle University. Arla® will provide milk and scientific support related to this nutritional intervention. This study is registered online at https://www.isrctn.com/ISRCTN13398279.

Data monitoring

Throughout the study, the principal investigator (AG) will monitor recruitment, retention and compliance figures with the core research team (AG, CH, LD, TA). The core team will meet regularly to plan and evaluate study’s day-to-day activities. Monthly meetings

For peer review only

will be organised with the co-investigators (KD, ES, AAS) to update on study management and progress. The core research team and co-investigators will prepare consents, assessments, study protocol, and standard operating procedures for: (1) assessments and data reporting; (2) data management; (3) adverse events management and reporting, and (4) staff health risk assessment and safety procedures.

Adverse events

This is a low risk study. There is a small chance of transient muscle soreness, gastrointestinal discomfort, metabolic changes, and change in appetite. The chief investigator (TA) is clinically trained to oversee the research process, and the research team is trained in health and safety procedures during data collection. Each participant will be closely monitored and asked about any adverse events occurring at home or in the gym. Any suspected adverse events will be reported to the chief investigator (TA), who will also offer clinical oversight of the study. Any serious adverse events, as evaluated by TA thought to be related to the intervention, will be reported immediately to the study sponsor and relevant ethics committee. Because of the low risk of adverse events, no independent Data Monitoring and Safety Committee will be appointed for this pilot study. The NHS indemnity insurance scheme will apply to cover the potential legal liability cover for harm to participants arising from the research. North Tyneside Council has the public and product liability cover for any potential harm arising from the fitness facility and equipment.

For peer review only

Data management

Data will be collected and managed in accordance with the EU General Data Protection Regulation (2018). At consent, participants will be assigned a unique study ID that will be used to pseudonymise primary research data collected from interviews and intervention. Identifiable data will be stored separately and will be accessible only to members of the research team who have additional research passport checks approved as part of their research role. Pseudonymised paper-based assessments will be double data

entered, and all study data will be stored on secure, fire-wall and password protected servers of Newcastle University for 5 years.

Data Statement

Technical appendix, statistical code, and dataset will be available from the AGE Research Group data manager.

DISCUSSION

Strengths and limitations

To our knowledge, this is the first pilot study examining the fidelity of a whole food (milk) combined with RE intervention in community-dwelling older adults living in the UK. The primary aims of the MIlkMAN pilot are to determine the feasibility and acceptability of the intervention in the community, and to provide essential data for planned future substantive research. The secondary aims are exploratory because the pilot lacks power to identify differences in physical functioning between the groups. However, the exploratory findings will be helpful in informing power calculations for the definitive study. The intervention will be conducted under the close supervision of a trained research team including an exercise physiologist and a health psychologist in a local gym with an easy access to older adults. Participants naïve to gym environment will benefit from the familiarisation with RE programme to encourage self-guided continued engagement in the community. A post-intervention interview in the pilot will include the collection of qualitative evidence on the barriers and motivators of community-based interventions. To our knowledge, only one study has investigated the barriers and drivers of compliance with protein-rich diets with RE interventions[56], and none has included views of older adults about what motivates their willingness and keenness to continue.

For peer review only

This study has several limitations, which will inform the development of the subsequent trial. Physical activity and exercise are consistently reported as positive influences on muscle mass and function in healthy older adults[16,57], whilst the evidence for positive effects of protein-rich foods above the effect of RE on muscle in older adults with adequate nutrition and activity levels has been more mixed[58,59]. There may be more benefit for protein supplementation with RE in those with muscle weakness and physical frailty[20]. As the MIlkMAN pilot will enrol 30 participants with relatively healthy muscle, the effect of the intervention is likely to be minimal. To achieve clinically meaningful differences between the groups and to examine the effect of milk above the effect of RE, a larger sample size, longer duration of the intervention, and the inclusion of older adults with reduced physical functioning or probable sarcopenia[4] will be necessary. Previous studies have reported difficulties in recruiting older adults with (probable) sarcopenia for various reasons, including the multi-faceted nature of muscle health, the variety of muscle-related clinical outcomes relevant to sarcopenia, and the lack of routine diagnosis of sarcopenia in clinical practice[60]. However, the universal acceptance of a sarcopenia definition[4,10] and cut-offs for sarcopenia components[4,10], the availability of sarcopenia screening tools[30] for a rapid assessment of sarcopenia, and wider use of GP surgeries (that routinely derive an electronic Frailty Index from data held in healthcare records[61]) for recruitment will increase the potential for enrolling appropriate participants to the larger trial. To reduce the risk of muscle injury, diabetes and exacerbation of any other health risks not covered by the exclusion criteria, this pilot will not recruit older adults with BMI >30. However, in the light of continued debate about the relationship between overweight/obesity and adverse health outcomes[62], and to maximise the recruitment, the substantive study will consider those with a BMI <35.

We hypothesise that the ratio of protein to fat in whole milk in combination with RE may be beneficial to ageing muscle and superior to skimmed milk for MPS, physical performance and muscle soreness after exercise as observed in younger adults[27-29]. To test this hypothesis and accurately quantify the differences across the groups in the future study,

For peer review only

a validated chromatographic analysis of amino acid[63] and fat content[64] in Arla Cravendale® milk will be necessary through the scientific support of Arla®.

The present study will use BIA to assess body composition in participants pre and post-intervention. Although BIA has been used widely to estimate lean body mass in community-dwelling older adults via validated prediction formulas[65], there are several limitations to the method, including low sensitivity to detect changes in muscle mass and the effect of hydration/ dehydration on the analysis[66]. Ultrasound has been proposed as another non-invasive, safe, and easy-to-use method suitable for longitudinal monitoring of muscle mass[67] with higher sensitivity compared with BIA. While it requires technical skills[67], this method may be an appropriate strategy to minimise the limitations associated with BIA to detect changes in muscle mass. In addition, muscle measurements assessed by ultrasound can be compared to anthropometric measures used to estimate RE-induced changes in muscle cross-sectional area, such as thigh circumference and a skinfold thickness[68], whilst keeping in mind the limitations of the method in older and obese adults[67].

To minimise participant burden, the present study will use GS as a measure of overall muscle strength and for minimisation to allow equal distribution of participants with low GS across the groups. However, a future definitive trial will include repeat assessment of 1RM via submaximal testing at baseline and post-intervention[69], to provide a more reliable and internally valid assessment of muscle strength. Repeat assessment of 1RM for all exercises prescribed in the RE programme will enable a more specific evaluation of muscle strength changes following the intervention period.

In summary, this is the first pilot study examining the feasibility and acceptability of whole compared with skimmed milk in combination with RE conducted in a local gym in community-dwelling older adults in the UK. Qualitative data will be collected to inform the future substantive trial, and allow better understanding of the barriers and facilitators of community-based intervention. This pilot study has low statistical power to detect changes in physical functioning between the groups, however, the results will be used to aid the

For peer review only

development and refinement of a future clinical trial, including study design, power calculations, recruitment strategy, inclusion and exclusion criteria, and outcome measures.

Acknowledgements The authors would like to thank the North East and North Cumbria Clinical Research Network.

Funding This project is funded by the National Institute for Health Research (NIHR) Newcastle Biomedical Research Centre (reference number: BH Ref 173606 / PDB053), Newcastle University and supported by Arla® (in-kind (milk) and scientific advice about nutrition).

Author contribution AG, KD, ES, AAS, TA, LD, and CH developed and refined the study protocol. AG, KD, ES, AAS, and TA were responsible for study conception and design. AG drafted the manuscript. All co-authors revised the manuscript draft. AG was responsible for the analysis plan. All authors were responsible for critical revision and approved the final version of the manuscript.

Funding This project is funded by the National Institute for Health Research (NIHR) Newcastle Biomedical Research Centre (reference number: BH Ref 173606 / PDB053), Newcastle University and supported by Arla® (in-kind milk contribution).

Disclaimer The views expressed are those of the authors and not necessarily those of the NHS or NIHR.

Competing interest This study received 'in-kind' contribution from Arla®.

Ethics approval The North East – Newcastle and North Tyneside Research Ethic Committee 1 (REC reference number: 18/NE/0265).

Provenance and peer review Not commissioned; internally peer reviewed

Open Access This is an open access article distributed in accordance with the Creative Commons Attribution Non Commercial (CC BY-NC 4.0) license, which permits others to distribute, remix, adapt, build upon this work non-commercially, and license their derivative works on different terms, provided that the original work is properly cited, appropriate credit is given, and changes made indicated, and the use is non-commercial. See: http://creaticecommons.org/license/by/4.0/

For peer review only

REFERENCES

1. Office for National Statistics. *Population Estimates for UK, England and Wales, Scotland and Northern Ireland: mid-2016.* https://www.ons.gov.uk/peoplepopulationandcommunity/populationandmigration/populationestimates/bulletins/annualmidyearpopulationestimates/mid2017 (accessed on 10 January 2019).
2. Rowe JW, Kahn RL. Successful aging 2.0: conceptual expansions for the 21st century. *J Gerontol B Psychol Sci Soc Sci* 2015;70:593–6.
3. Drewnowski A, Evans WJ. Nutrition, physical activity, and quality of life in older adults: summary. *J Gerontol A Biol Sci Med Sci* 2001;56:89–94.
4. Cruz-Jentoft AJ, Bahat G, Bauer J, et al. Sarcopenia: revised European consensus on definition and diagnosis. *Age Ageing* 2018 Oct 12. doi: 10.1093/ageing/afy169. [Epub ahead of print]
5. Janssen I, Ross R. Linking age-related changes in skeletal muscle mass and composition with metabolism and disease. *J Nutr Health Aging* 2005;9:408–19.
6. Visser, M.; Schaap, L.A. Consequences of sarcopenia. *Clin Geriatr Med* 2011;27: 387–399.
7. Manrique-Espinoza B, Salinas-Rodríguez, A, et al. Sarcopenia is associated with physical and mental components of health-related quality of life in older adults. *J Am Med Dir Assoc* 2017;18:636.e1–636.e5.
8. Mithal A, Bonjour JP, Boonen, S, et al. Impact of nutrition on muscle mass, strength, and performance in older adults. *Osteoporos Int* 2013;24:1555–66.
9. Marzetti E, Calvani R, Tosato M, et al. Physical activity and exercise as countermeasures to physical frailty and sarcopenia. *Aging Clin Exp Res* 2017;29: 35–42.
10. Cruz-Jentoft AJ, Sayer AA. Sarcopenia. *Lancet* 2019;393:2636–46.
11. Cruz-Jentoft AJ, Landi F, Schneider SM, et al. Prevalence of and interventions for sarcopenia in ageing adults: a systematic review. Report of the International Sarcopenia Initiative (EWGSOP and IWGS). *Age Ageing* 2014;43:748–59.
12. Pinedo-Villanueva R, Westbury LD, Syddall HE, et al. Health care costs associated with muscle weakness: A UK population-based estimate. *Calcif Tissue Int* 2018 Sep 22. doi: 10.1007/s00223-018-0478-1. [Epub ahead of print]
13. Volaklis KA, Halle M, Thorand B, et al. Handgrip strength is inversely and independently associated with multimorbidity among older women: Results from the KORA-Age study. *Eur J Intern Med* 2016, 31, 35–40.
14. Tsekoura M, Kastrinis A, Katsoulaki M, Billis E, Gliatis J. Sarcopenia and its impact on quality of life. *Adv Exp Med Biol* 2017;987:213–8.
15. Churchward-Venne TA, Holwerda AM, Phillips SM, et al. What is the optimal amount of protein to support post-exercise skeletal muscle reconditioning in the older adult? *Sports Med* 2016;46:1205–12.
16. Peterson M, Sen A, Gordon P. Influence of resistance exercise on lean body mass in aging adults: a meta-analysis. *Med Sci Sports Exerc* 2011;43:249–58.
17. Pennings B, Koopman R, Beelen M, et al. Exercising before protein intake allows for greater use of dietary protein–derived amino acids for de novo muscle protein synthesis in both young and elderly men. *Am J Clin Nutr* 2011;93:322–31.
18. Witard OC, Jackman SR, Breen L, et al. Myofibrillar muscle protein synthesis rates subsequent to a meal in response to increasing doses of whey protein at rest and after resistance exercise. *Am J Clin Nutr* 2014;99:86–95.

For peer review only

19. Moore DR, Churchward-Venne TA, Witard O, et al. Protein ingestion to stimulate myofibrillar protein synthesis requires greater relative protein intakes in healthy older versus younger men. *J Gerontol A Biol Sci Med Sci* 2015;70:57–62.
20. Tieland M, Dirks ML, van der Zwaluw N, et al. Protein supplementation increases muscle mass gain during prolonged resistance-type exercise training in frail elderly people: a randomized, double-blind, placebo-controlled trial. *J Am Med Dir Assoc* 2012;13:713–9.
21. Millward DJ. Nutrition and sarcopenia: evidence for an interaction. *Proc Nutr Soc* 2012;71:566–75.
22. Appleton KM. Barriers to and facilitators of the consumption of animal-based protein-rich foods in older adults. *Nutrients* 2016;8:187.
23. Pereira PC. Milk nutritional composition and its role in human health. *Nutrition* 2014;30:619–27.
24. Naclerio F, Larumbe-Zabala E. Effects of whey protein alone or as part of a multi-ingredient formulation on strength, fat-free mass, or lean body mass in resistance-trained individuals: a meta-analysis. *Sports Med* 2016;46:125–37.
25. Tang JE, Moore DR, Kujbida GW, Tarnopolsky MA, Phillips SM. Ingestion of whey hydrolysate, casein, or soy protein isolate: effects on mixed muscle protein synthesis at rest and following resistance exercise in young men. *J Appl Physiol (1985)* 2009;107:987–92.
26. Yang Y, Breen L, Burd NA, et al. Resistance exercise enhances myofibrillar protein synthesis with graded intakes of whey protein in older men. *Br J Nutr* 2012;108:1780–8.
27. Elliot TA, Cree MG, Sanford AP, et al. Milk ingestion stimulates net muscle protein synthesis following resistance exercise. *Med Sci Sports Exerc* 2006;38:667–74.
28. Rankin P, Stevenson EJ, Cockburn E. The effect of milk on the attenuation of exercise-induced muscle damage in males and females. *Eur J Appl Physiol* 2015;115:1245–61.
29. Rankin P, Landy A, Stevenson E, et al. Milk: an effective recovery drink for female athletes. *Nutrients* 2018;10(2).
30. Malmstrom TK, Morley JE. SARC-F: a simple questionnaire to rapidly diagnose sarcopenia. *J Am Med Dir Assoc* 2013;14:531–2.
31. Cruz-Jentoft AJ, Baeyens JP, Bauer JM, et al.; European Working Group on Sarcopenia in Older People. Sarcopenia: European consensus on definition and diagnosis: Report of the European Working Group on Sarcopenia in Older People. *Age Ageing* 2010;39:412–23.
32. Guralnik JM, Simonsick EM, Ferrucci L, et al. A short physical performance battery assessing lower extremity function: association with self-reported disability and prediction of mortality and nursing home admission. *J Gerontol* 1994;49:M85–94.
33. Moon JR, Stout JR, Smith-Ryan AE, et al. Tracking fat-free mass changes in elderly men and women using single-frequency bioimpedance and dual-energy X-ray absorptiometry: a four-compartment model comparison. *Eur J Clin Nutr* 2013;67 Suppl 1:S40–6.
34. Roberts HC, Denison HJ, Martin HJ, et al. A review of the measurement of grip strength in clinical and epidemiological studies: towards a standardised approach. *Age Ageing* 2011;40:423–9.

For peer review only

35. Gandek B, Ware JE, Aaronson NK, et al. Cross-validation of item selection and scoring for the SF-12 Health Survey in nine countries: results from the IQOLA Project. International Quality of Life Assessment. *J Clin Epidemiol* 1998;51:1171–8.
36. Wade DT, Collin C. The Barthel ADL Index: a standard measure of physical disability? *Int Disabil Stud* 1988;10:64–7.
37. Saghaei, M, Saghaei, S. Implementation of an open-source customizable minimization program for allocation of patients to parallel groups in clinical trials. *J Biomed Sci Eng* 2011;4:734–9.
38. Moore CG, Carter RE, Nietert PJ, Stewart PW. Recommendations for planning pilot studies in clinical and translational research. *Clin Transl Sci* 2011;4(5):332-7.
39. Chandola T, Jenkinson C. The new UK National Statistics Socio-Economic Classification (NS-SEC); investigating social class differences in self-reported health status. *J Public Health Med* 2000;22:182–90.
40. Smith T, Noble M, Noble S, et al. *The English Indices of Deprivation 2015*. London: Department of Communities and Local Government; 2015. https://assets.publishing.service.gov.uk/government/uploads/system/uploads/attachment_data/file/464485/English_Indices_of_Deprivation_2015_-_Technical-Report.pdf. Accessed April 12, 2018.
41. Brown LM, Schinka JA. Development and initial validation of a 15-item informant version of the Geriatric Depression Scale. *Int J Geriatr Psychiatry* 2005;20:911–8.
42. Simpson E, Bradley J, Poliakov I, et al. Iterative development of an online dietary recall tool: INTAKE24. *Nutrients* 2017;9(2).
43. Wilson MM, Thomas DR, Rubenstein LZ, et al. Appetite assessment: simple appetite questionnaire predicts weight loss in community-dwelling adults and nursing home residents. *Am J Clin Nutr* 2005;82:1074–81.
44. Collerton J, Barrass K, Bond J, et al. The Newcastle 85+ study: biological, clinical and psychological factors associated with healthy ageing: study protocol. *BMC Geriatr* 2007;7:14.
45. Robinson MM, Dasari S, Konopka AR, et al. Enhanced protein translation underlies improved metabolic and physical adaptations to different exercise training modes in young and old humans. *Cell Metab* 2017;25:581–92.
46. Peterson MD, Gordon PM. Resistance exercise for the aging adult: clinical implications and prescription guidelines. *Am J Med* 2011;124:194–8.
47. Borde R, Hortobágyi T, Granacher U. Dose-response relationships of resistance training in healthy old adults: a systematic review and meta-analysis. *Sports Med* 2015;45:1693–720.
48. Brzycki, M. Strength testing—predicting a one-rep max from reps-to-fatigue. *J Phys Educ Recreat Dance* 1993;64:88–90.
49. Borg E, Borg G. A comparison of AME and CR100 for scaling perceived exertion. *Acta Psychol (Amst)* 2002;109:157–5.
50. Hurst C, Weston KL, Weston M. The effect of 12 weeks of combined upper-and lower-body high-intensity interval training on muscular and cardiorespiratory fitness in older adults. *Aging Clin Exp Res* 2019;31:661–71.
51. Whelton PK, Carey RM, Aronow WS, et al. 2017 ACC/AHA/AAPA/ABC/ACPM/AGS/APhA/ASH/ASPC/NMA/PCNA Guideline for the Prevention, Detection, Evaluation, and Management of High Blood Pressure in Adults: Executive Summary: A Report of the American College of

For peer review only

Cardiology/American Heart Association Task Force on Clinical Practice Guidelines. *Circulation* 2018;138:e426–83.
52. Rezk CC, Marrache RC, Tinucci T, et al. Post-resistance exercise hypotension, hemodynamics, and heart rate variability: influence of exercise intensity. *Eur J Appl Physiol* 2006;98:105–12.
53. Huggett DL, Elliott ID, Overend TJ, et al. Comparison of heart-rate and blood-pressure increases during isokinetic eccentric versus isometric exercise in older adults. *J Aging Phys Act* 2004;12:157–69.
54. The Food Standards Agency (2002). *McCance and Widdowson's the Composition of Foods Integral Dataset* (6th Summary Edition). http://webarchive.nationalarchives.gov.uk. Accessed in: August 20, 2017
55. Hsieh HF, Shannon SE. Three approaches to qualitative content analysis. *Qual Health Res* 2005;15:1277–88.
56. Herrema AL, Westerman, MJ, van Dongen EJI, et al. Combined protein-rich diet with resistance exercise intervention to counteract sarcopenia: a qualitative study on drivers and barriers of compliance. *J Aging Phys Act* 2018;26:106–13.
57. Beaudart C, Dawson A, Shaw SC, et al. Nutrition and physical activity in the prevention and treatment of sarcopenia: systematic review. *Osteoporos Int* 2017;28:1817–33.
58. Daly RM, O'Connell SL, Mundell NL, et al. Protein-enriched diet, with the use of lean red meat, combined with progressive resistance training enhances lean tissue mass and muscle strength and reduces circulating IL-6 concentrations in elderly women: a cluster randomized controlled trial. *Am J Clin Nutr* 2014;99:899–910.
59. Kukuljan S, Nowson CA, Sanders K, et al. Effects of resistance exercise and fortified milk on skeletal muscle mass, muscle size, and functional performance in middle-aged and older men: an 18-mo randomized controlled trial. *J Appl Physiol* 2009;107:1864–73.
60. Edwards MH, Dennison EM, Aihie Sayer A, et al. Osteoporosis and sarcopenia in older age. *Bone* 2015;80:126–30.
61. Clegg A, Bates C, Young J, et al. Development and validation of an electronic frailty index using routine primary care electronic health record data. *Age Ageing* 2016;45:353–60.
62. Flegal KM, Ioannidis JPA, Doehner W. Flawed methods and inappropriate conclusions for health policy on overweight and obesity: the Global BMI Mortality Collaboration meta-analysis. *J Cachexia Sarcopenia Muscle* 2019;10:9–13.
63. Otter DE. Standardised methods for amino acid analysis of food. *Br J Nutr* 2012;108 Suppl 2:S230–7.
64. Amores G, Virto M. Total and free fatty acids analysis in milk and dairy fat. *Separations* 2019;6:14. doi:10.3390/separations6010014.
65. Scafoglieri A, Clarys JP, Bauer JM, et al. Predicting appendicular lean and fat mass with bioelectrical impedance analysis in older adults with physical function decline - The PROVIDE study. *Clin Nutr* 2017;36:869–75.
66. Thompson DL, Thompson WR, Prestridge TJ, et al. Effects of hydration and dehydration on body composition analysis: a comparative study of bioelectric impedance analysis and hydrodensitometry. *J Sports Med Phys Fitness* 1991;31:565–70.

For peer review only

67. Heymsfield SB, Gonzalez MC, Lu J, et al. Skeletal muscle mass and quality: evolution of modern measurement concepts in the context of sarcopenia. Proc Nutr Soc 2015;74:355–66.
68. DeFreitas JM, Beck TW, Stock MS, et al. A comparison of techniques for estimating training-induced changes in muscle cross-sectional area. *J Strength Cond Res* 2010;24:2383–9.
69. Hurst C, Batterham AM, Weston KL, et al. Short-and long-term reliability of leg extensor power measurement in middle-aged and older adults. *J Sports Sci* 2018;36:970–7.

Figure legend

Figure 1. Study flow chart.

The recruitment (assessment of eligibility) up to allocation (randomisation into 3 groups) will be finalised within 3 months, followed by baseline assessments for health and functioning in participants' homes, and a 6-week intervention in a local gym. A post-intervention assessment will be conducted over 3 weeks in participants' homes. Data will be analysed following data entry, cleaning, and quality assurance over 2-3 months.

For peer review only

For peer review only

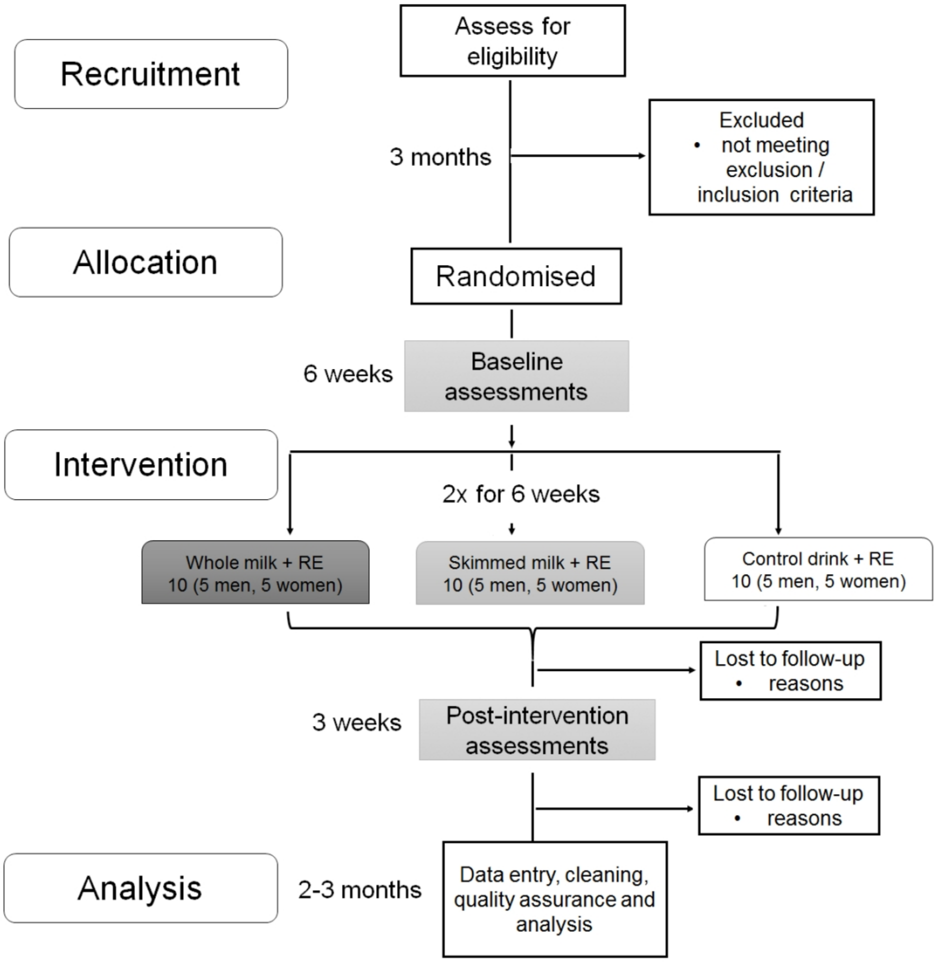

Figure 1. Study flow chart.

109x111mm (300 x 300 DPI)

SUPPLEMENTARY MATERIAL

Appendix 1

THE SARC-F QUESTIONNAIRE[30]

Strength: How much difficulty do you have in lifting and carrying 10 pounds / a bag of shopping?
None: 0
Some: 1
A lot or unable: 2

Assistance in walking: How much difficulty do you have walking across a room?
None: 0
Some: 1
A lot, use aids, or unable: 2

Rise from a chair: How much difficulty do you have transferring from a chair or bed?
None: 0
Some: 1
A lot or unable without help: 2

Climb stairs: How much difficulty do you have climbing a flight of 10 stairs?
None: 0
Some: 1
A lot or unable: 2

Falls: How many times have you fallen in the past year?
None: 0
1 to 3 falls: 1
4 or more falls: 2

For peer review only

Appendix 2

CR100 scale[49]

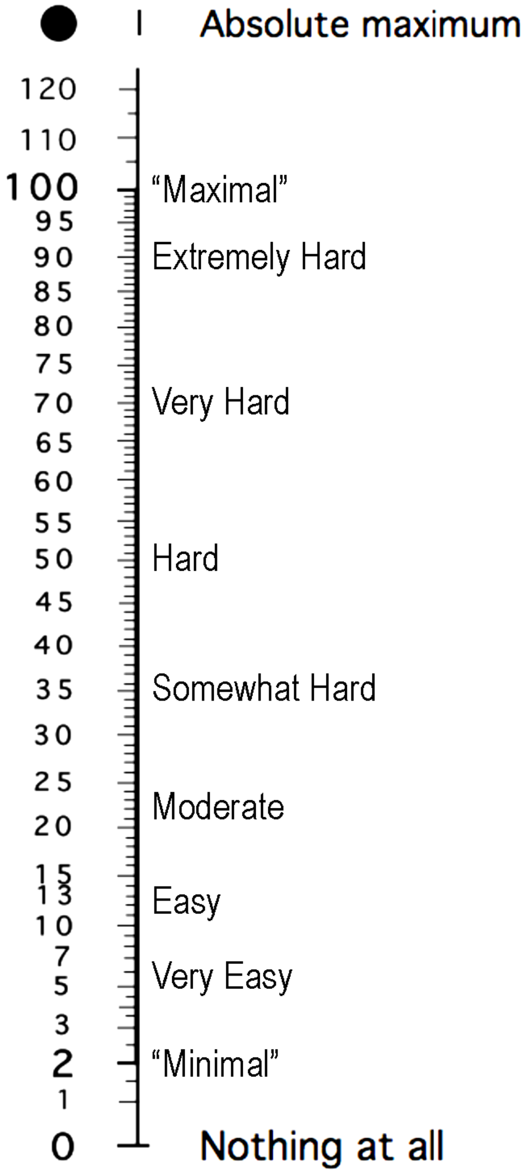

Instructions for participants (explained to participants by exercise physiologist)
Assessment ~10 minutes after exercise

- Using the scale, we would like you to rate your perceptions of EFFORT (RPE), that is, how difficult the session felt to you.
- Your perception of EFFORT should be a conscious awareness of how hard (or easy) the whole session was. It should not be influenced your feelings of fatigue, pain or discomfort (try separating these from effort as best you can).
- You will be asked to rate your perceptions of overall effort (the whole session after the warm-up), and then separate scores for upper-body muscle effort (arm muscles) and lower-body muscle effort (leg muscles).

Appendix 3

Visual analogue scale for muscle soreness

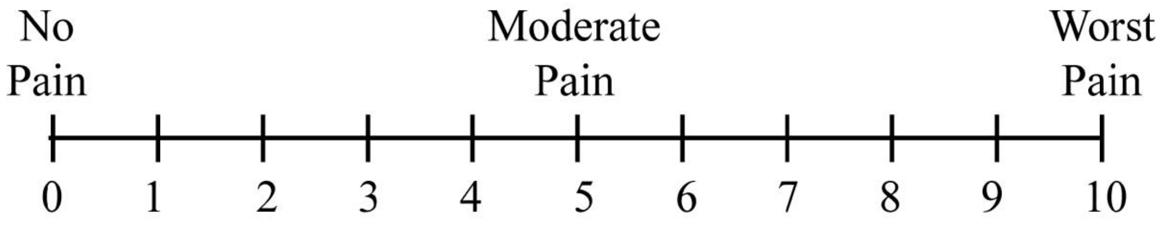

0 = no pain, 1-3 = mild pain, 4-6 = moderate pain; 7-10 = severe

Instructions for participants (explained to participants by a researcher)

- This is a Visual Analogue Scale. The scale describes the intensity of your muscle soreness, 0 meaning no pain at all, 1 to 3 meaning mild pain, 4-6 moderate pain, and 7-10 the worst pain.
- Assessment ~45 minutes after exercise: Which number on the scale describes the best your muscle soreness in your arms and legs?
- *Assessment in the evening over the telephone (6-7 hours after exercise)*: A researcher will call you in the evening after each visit to the gym to ask you again about your muscle soreness in your arms and legs. Please use this visual analogue scale to rate your muscle soreness in your arms and legs.

For peer review only
